# Tetramerization of deoxyadenosine kinase meets the demands of a DNA replication substrate challenge in *Giardia intestinalis*

**DOI:** 10.1093/nar/gkae1073

**Published:** 2024-11-28

**Authors:** Farahnaz Ranjbarian, Karim Rafie, Kasturika Shankar, Sascha Krakovka, Staffan G Svärd, Lars-Anders Carlson, Anders Hofer

**Affiliations:** Department of Medical Biochemistry and Biophysics, Umeå University, Linnaeus väg 6, SE-901 87 Umeå, Sweden; Umeå Centre for Microbial Research (UCMR), Umeå University, Linnaeus väg 6, SE-901 87 Umeå, Sweden; Department of Medical Biochemistry and Biophysics, Umeå University, Linnaeus väg 6, SE-901 87 Umeå, Sweden; Umeå Centre for Microbial Research (UCMR), Umeå University, Linnaeus väg 6, SE-901 87 Umeå, Sweden; Laboratory for Molecular Infection Medicine Sweden (MIMS), Umeå University, Linnaeus väg 6, SE-901 87 Umeå, Sweden; Wallenberg Centre for Molecular Medicine (WCMM), Umeå University, Linnaeus väg 6, SE-901 87 Umeå, Sweden; Department of Molecular Pharmacology, Groningen Research Institute of Pharmacy (GRIP), University of Groningen, Deusinglaan 1, 9713 AVGroningen, The Netherlands; Department of Medical Biochemistry and Biophysics, Umeå University, Linnaeus väg 6, SE-901 87 Umeå, Sweden; Umeå Centre for Microbial Research (UCMR), Umeå University, Linnaeus väg 6, SE-901 87 Umeå, Sweden; Laboratory for Molecular Infection Medicine Sweden (MIMS), Umeå University, Linnaeus väg 6, SE-901 87 Umeå, Sweden; Wallenberg Centre for Molecular Medicine (WCMM), Umeå University, Linnaeus väg 6, SE-901 87 Umeå, Sweden; Department of Cell and Molecular Biology, Uppsala University, Husargatan 6, BMC Box 596, SE-75124 Uppsala, Sweden; Department of Cell and Molecular Biology, Uppsala University, Husargatan 6, BMC Box 596, SE-75124 Uppsala, Sweden; Department of Medical Biochemistry and Biophysics, Umeå University, Linnaeus väg 6, SE-901 87 Umeå, Sweden; Umeå Centre for Microbial Research (UCMR), Umeå University, Linnaeus väg 6, SE-901 87 Umeå, Sweden; Laboratory for Molecular Infection Medicine Sweden (MIMS), Umeå University, Linnaeus väg 6, SE-901 87 Umeå, Sweden; Wallenberg Centre for Molecular Medicine (WCMM), Umeå University, Linnaeus väg 6, SE-901 87 Umeå, Sweden; Department of Medical Biochemistry and Biophysics, Umeå University, Linnaeus väg 6, SE-901 87 Umeå, Sweden; Umeå Centre for Microbial Research (UCMR), Umeå University, Linnaeus väg 6, SE-901 87 Umeå, Sweden

## Abstract

The protozoan parasite *Giardia intestinalis* is one of only a few organisms lacking *de novo* synthesis of DNA building blocks (deoxyribonucleotides). Instead, the parasite relies exclusively on salvaging deoxyadenosine and other deoxyribonucleosides from its host environment. Here, we report that *G. intestinalis* has a deoxyribonucleoside kinase with a 1000-fold higher catalytic efficiency (k_cat_/K_M_) for deoxyadenosine than the corresponding mammalian kinases and can thereby provide sufficient deoxyadenosine triphosphate levels for DNA synthesis despite the lack of *de novo* synthesis. Several deoxyadenosine analogs were also potent substrates and showed comparable EC_50_ values on cultured *G. intestinalis* cells as metronidazole, the current first-line treatment, with the additional advantage of being effective against metronidazole-resistant parasites. Structural analysis using cryo-EM and X-ray crystallography showed that the enzyme is unique within its family of deoxyribonucleoside kinases by forming a tetramer stabilized by extended N- and C-termini in a novel dimer–dimer interaction. Removal of the two termini resulted in lost ability to form tetramers and a markedly reduced affinity for the deoxyribonucleoside substrate. The development of highly efficient deoxyribonucleoside kinases via oligomerization may represent a critical evolutionary adaptation in organisms that rely solely on deoxyribonucleoside salvage.

## Graphical Abstract

**Graphical Abstract:**
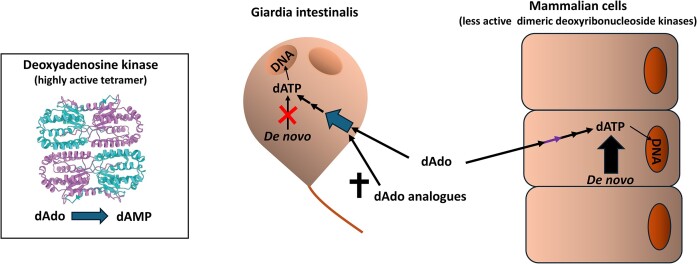

## Introduction

The protozoan parasite *Giardia intestinalis* (synonymous to *Giardia lamblia* and *Giardia duodenalis*) causes giardiasis, which is an acute or chronic diarrheal disease spread by contaminated water or food and leads to 190 million symptomatic cases per year (1,2). However, the disease is also associated with a wide range of other symptoms, including stomach cramps, vomiting, nausea, flatulence, dehydration, malabsorption, arthritis and chronic fatigue syndrome. It can also start out as an asymptomatic infection but still cause malabsorption in children, followed by growth retardation, suppressed cognitive development and sometimes death. Metronidazole (Flagyl) is the most commonly used drug to treat giardiasis and selectively kills the parasite and other anaerobic organisms by forming free radicals under oxygen-limited conditions, but it has side effects such as nausea, abdominal pain, diarrhea, and in some cases, neurotoxicity reactions (3). Drug resistance to metronidazole and other 5-nitroimidazoles is emerging (4), highlighting the need to develop new drugs.

*Giardia intestinalis* is a diplomonad, which is a group of flagellated organisms characterized by having a duplicated cell content with two diploid nuclei in each cell (1). The parasite has two life cycle stages, trophozoites and cysts. The trophozoite is the replicating form and attaches to the surface of epithelial cells in the duodenum where it proliferates and lives on the ingested food of the host. The trophozoites can also secrete material to form a cyst wall and go through two rounds of DNA replication to form cysts, which contain four nuclei and a 16N genome per cell (4N in each nucleus). The cysts, which leave the body in the feces, have only limited metabolic activity and are very resistant to diverse environmental factors. This makes them well adapted for survival outside the body for extended periods. When ingested, a combination of the acid pH in the stomach and the subsequent exposure to trypsin and bile in the duodenum induce parasite excystation into the replicating trophozoite form (5).

**Figure 1.**
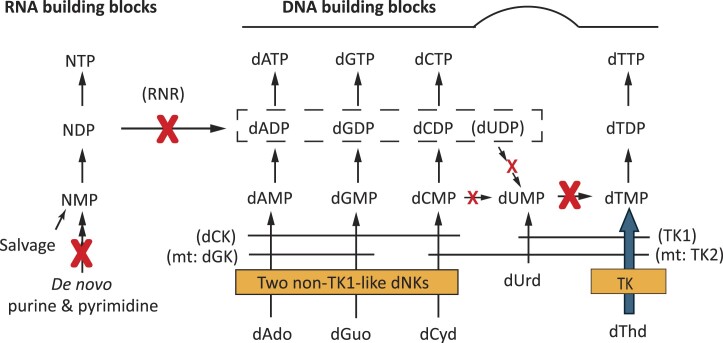
*Giardia intestinalis* nucleotide metabolism. Key reactions/pathways lacking in the parasite are marked with large crosses and other missing reactions with smaller crosses. Of particular importance for this work is the lack of ribonucleotide reductase (RNR), which is a key enzyme for the synthesis of DNA building blocks by producing deoxyribonucleoside diphosphates (shown in the hatched square) from the corresponding ribonucleoside diphosphates (NDPs). The lack of RNR (as well as thymidylate synthase) makes it necessary for the parasite to use preformed deoxyribonucleosides, which are phosphorylated by dNKs (orange squares). For comparison, mammalian cells have RNR as well as cytosolic dNKs and mitochondrial (mt) dNKs with substrate specificities indicated by horizontal lines. Studies of the *G. intestinalis* thymidine kinase (TK) show that it has an unusually high substrate affinity (indicated by a thick arrow), which helps the parasite to efficiently compete with the mammalian cells for thymidine. The properties of the two non-TK1-like dNKs are not yet known, but it is likely that the three indicated substrates must be phosphorylated in order to provide all dNTPs for DNA synthesis in the parasite.

It is common that protozoan parasites lack *de novo* purine biosynthesis, which makes them sensitive to drugs targeting their compensatory salvage pathways needed to take up and use purines from the surroundings (6–9). The drugs can either be inhibitors of the salvage process or more commonly act as substrate analogs. In the latter case, the salvage enzymes convert them to their nucleotide form and inhibit downstream processes such as nucleic acid synthesis, energy metabolism or other nucleotide-dependent reactions. In the case of *G. intestinalis*, it is not only *de novo* purine biosynthesis that is lacking. In fact, the parasite lacks *de novo* synthesis of all nucleotides including the two main pathways to synthesize purines and pyrimidines, as well as the additional reactions needed to make deoxyribonucleotides and thymidylate (10–12). It is particularly striking that it lacks ribonucleotide reductase (RNR in Figure 1), which is a key enzyme for *de novo* synthesis of deoxyribonucleoside triphosphates (dNTPs) needed as building blocks for DNA (13). Only a few organisms lack this enzyme, including *Giardia, Ureaplasma* as well as some species of *Entamoeba* and *Borrelia* (12,14–16). The lack of RNR makes *G. intestinalis* and other *Giardia* species completely dependent on salvaging deoxyribonucleosides from the surroundings to make the corresponding dNTPs. *Giardia* trophozoites secrete an extracellular nuclease upon contact with host cells (17), potentially assisting the parasite in digesting DNA from our food as a source of deoxyribonucleosides. It is not exactly known how the deoxyribonucleosides are taken up, but according to the limited information available, *G. intestinalis* has one type of transporter for oxopyrimidine nucleosides and a second one with general nucleoside specificity (18). Both of them are able to recognize ribonucleosides as well as deoxyribonucleosides.

The first intracellular step in the salvage process is to phosphorylate the deoxyribonucleosides to the monophosphate form (Figure 1). The reaction is catalyzed by deoxyribonucleoside kinases (dNKs), which is considered the rate-limiting phosphorylation step in dNTP synthesis (19). The dNKs are subdivided into two structurally unrelated families, the thymidine kinase 1 (TK1) family and a family containing the remaining enzymes referred to as non-TK1-like dNKs (20). The non-TK1-like dNKs are further subdivided into a monophyletic group of canonical non-TK1-like dNKs and a second group with TKs from *Herpesviridae*, which are structurally related to the canonical group but share very little amino acid sequence homology. The TK1 family has a strict substrate specificity adapted to phosphorylate thymidine-like substrates (thymidine and deoxyuridine) and serve the purpose to supply the cell with dTTP (20). The TK from *G. intestinalis* was recently characterized and this TK1-like enzyme was found to have a ∼10-fold lower K_M_ value for thymidine than the human TK1 (21). The high substrate affinity could be an adaptation of *G. intestinalis* to effectively compete with the host cells for scarce thymidine supplies and thereby compensates for the lack of *de novo* dTTP synthesis (Figure 1). The high affinity of the enzyme also makes the parasite sensitive to the substrate analog azidothymidine, which is a potent antigiardial agent in *in vitro* cell proliferation assays as well as in *G. intestinalis*-infected gerbils (21). The incentive behind the current study was to investigate if also other dNKs in *G. intestinalis* have developed higher substrate affinities than the host enzymes as an adaptation to survive in the mammalian body. Studies of *G. intestinalis*, *T. brucei* and mammalian TK1 members have shown that substrate affinity can be gained by either increasing the number of subunits or domains that the enzyme is composed of (7,21–23). However, it has yet not been demonstrated for the non-TK1-like dNKs, which are, in most cases, dimers (19).

The non-TK1-like family of dNKs generally have much broader substrate specificities than the TK1 family. Mammalian cells have a full capacity to phosphorylate deoxyribonucleosides in the cytosol as well as the mitochondria and the single cytosolic member of this family, deoxycytidine kinase (dCK), is able to phosphorylate deoxycytidine, deoxyadenosine and deoxyguanosine (19). In contrast, *G. intestinalis* lacks mitochondria and only needs cytosolic dNKs. Besides the TK mentioned above, the parasite also has two non-TK1-like dNKs. Very little is known about them. A previous study showed that the dNK activities from a *G. intestinalis* extract separated as two peaks during purification, one for purine substrates and one for pyrimidines (24). This does not match the current knowledge that there are three dNKs encoded in the genome whereof the one studied is completely specific for thymidine. The lack of knowledge about these essential enzymes in the parasite has hampered the understanding of *Giardia* deoxyribonucleoside metabolism and hence its exploitation as a target for antiparasitic drugs.

In the current work, we have characterized one of the two non-TK1-like dNKs from *G. intestinalis*. The enzyme was found to use deoxyadenosine as its main substrate and was consequently given the name deoxyadenosine kinase (dAK). Several analogs of deoxyadenosine were also recognized as substrates, and cell studies showed that these analogs seem to be promising as drug candidates against the parasite. Structural analysis showed that *G. intestinalis* dAK is a homotetramer, which dramatically loses substrate affinity when tetramer formation is prevented by removal of the extended N- and C-termini at the dimer–dimer interface. For an organism that solely survives on deoxyribonucleosides for the synthesis of dNTPs, it is of utmost importance to efficiently salvage them from the surroundings in the host. Increasing the number of subunits that the dAK consists of may have been a fast-track evolutionary pathway of the *Giardia* genus to dramatically increase the substrate affinity and make itself independent of *de novo* synthesis of dATP.

## Materials and methods

### Amino acid sequence alignment

The alignment was performed by the DNA Star Lasergene 17 software using the Clustal W algorithm. The dNKs used for the alignment were *Homo sapiens* dCK, deoxyguanosine kinase (dGK) and TK2 (NP_000779.1, NP_550438.1 and TK2 AAC51167.1, respectively), *Drosophila melongaster* dNK (NP_001262722.1), *Lactobacillus acidophilus* dAK (AAB09750.1), *Bacillus cereus* dAK (KLA31806.1), and *Mycoplasma mycoides* dAK. The two non-TK1-like dNKs from *G. intestinalis*, dNK2 (unknown substrate specificity) and dAK were taken from GiardiaDB (https://giardiadb.org) assemblage A isolate WB genome sequenced in 2019 (gene numbers GL50803-004558 and GL50803-0017451, respectively). The *G. intestinalis*WB isolate is also referred to as WB/C6.

### Cloning of the *G. intestinalis* dAK into an expression vector

The identified *G. intestinalis* dAK gene in the assemblage A isolate WB (GL50803-17451 at https://giardiadb.org/giardiadb) was amplified with polymerase chain reaction (PCR) using extracted genomic DNA from the *G. intestinalis* isolate WB as template. The PCR reaction was performed with the primers GT**C CAT GG**C TGC GCG AAA TCT GG (forward primer) and CAT GCT **GGT ACC** TTA ATA GAA ATT GGA AGC TGA TTG CG (reverse primer) with included restriction sites marked in boldface for NcoI and Acc65I, respectively. The PCR-amplified gene was cloned into the pETZ vector from European Molecular Biology Laboratory for expression in *Escherichia coli*. The resulting plasmid construct (pETZ-dAK) encoded an N-terminal His_6_-tagged fusion partner Z (10 kDa IgG-binding domain protein) followed by a tobacco etch virus (TEV) cleavage site connected to the 29 kDa dAK. The obtained clones were verified to be correct by sequencing. Similar experiments were also performed to make truncated versions of the protein: dAK-ΔN (amino acids 30–251), dAK-ΔC (amino acids 1–232) and dAK-ΔNΔC (amino acids 30–232). The forward primer was then substituted by GTG **CCA TGG** CAA AGA TGT TCA TCT CTA TAT CAG G for the removal of the N-terminus and the reverse primer was substituted by GTC AGA **GGT ACC** TTA GTC TAC GAT GGC TTG GTG TTT C for the removal of the C-terminus. Both primers were substituted for the construction of the mutant lacking both termini.

### Protein expression and purification

The pETZ-dAK vector was introduced into the *E. coli* strain BL21(DE3)plys for recombinant expression of the *G. intestinalis* dAK. The bacteria were cultivated in LB medium supplemented with 50 μg/ml kanamycin and 34 μg/ml chloramphenicol at 37°C. Protein expression was induced at OD_600_ ≃ 0.6 with 0.5 mM isopropyl β-d-thiogalactopyranoside for overnight induction at 18°C. The cells were harvested by centrifugation at 4000 × *g* for 15 min. The pellet was washed with 20 mM Tris-HCl (pH = 7.5), and re-centrifuged at 4000 × *g* for 10 min. The supernatant was discarded, and the pellet was resuspended in lysis buffer [20 mM Tris-HCl (pH = 7.5), 0.4 M NaCl and 0.1 mM phenylmethylsulfonide fluoride] using a volume of 10 ml per g of pellet. The suspension was frozen and thawed, sonicated and centrifuged at 40 000 rpm for 45 min (Beckman L-90 ultracentrifuge using a Ti70 rotor). The supernatant was loaded onto a 2 ml column containing Ni-NTA His·Bind resin (Merck). The column was first washed with a solution containing 20 mM Tris-HCl (pH = 7.5), 0.4 M NaCl and 20 mM imidazole. The second wash was with 50 mM imidazole (other buffer constituents were the same). Finally, the Z-tagged dAK was eluted with a solution containing 20 mM Tris-HCl (pH = 7.5), 150 mM NaCl and 250 mM imidazole. In order to remove the fusion partner, the protein was first exchanged into an imidazole-free buffer containing 20 mM Tris-HCl (pH = 7.5) and 150 mM NaCl. TEV protease was added to the Z-tagged dAK using a molar ratio of 1:10, and the proteins were incubated for 3 h at room temperature. The mixture was then loaded onto a 0.4 ml Ni-NTA His·Bind resin to bind the His_6_-tagged fusion partner and collect the *G. intestinalis* dAK in the flow-through. The protein was frozen in liquid nitrogen and stored at −80°C.

### Enzyme assay of dAK

*Giardia intestinalis* dAK was incubated in 50 μl of buffer containing 2 mM adenosine triphosphate (ATP), 5 mM MgCl_2_, 0.5 mM dithiothreitol (DTT), 100 mM potassium acetate and 50 mM Tris-HCl (pH = 7.5), with various concentrations of [2,8–^3^H]-deoxyadenosine at 37°C for 30 min. These standard conditions were based on optimization experiments (Supplementary Figure S1). After completion of the assay, the enzyme was inactivated by an incubation at 100°C for 2 min to stop the reaction. In this radioactive assay, the product was separated from the substrate on filters as described before (21) [modified version of previous protocol (25)]. Briefly, 20 μl of each reaction was spotted onto a DE-81 filter (Whatman), dried, washed three times for 5 min with 1 mM ammonium formate and finally incubated in 1 ml HCL/KCL (0.1 M of each) with shaking for 2 min before scintillation counting. For other substrates than deoxyadenosine, the product was separated from the substrate with high performance liquid chromatography (HPLC) analysis as described below.

### HPLC analysis

Nucleotides from enzyme assays (as well as the protein preparations in Supplementary Figure S2) were analyzed using a 150 mm × 4.6 mm SunShell C18-WP HPLC column from ChromaNik Technologies Inc (Osaka, Japan) using a modified version of a previous published protocol developed for a multisubstrate assay with all five substrates (dCyd, dUrd, dThd, dGuo and dAdo) (21). Instead of using a ternary mixture of three aqueous solutions, we changed it to a binary mixture of A and B, with the third component, tetrabuytylammonium bromide (TBA-Br) mixed into the two other solutions instead of using it separately. Solution A contained 0.7 g/l TBA-Br, 7% (*v/v*) methanol and 23 g/l KH_2_PO_4_, adjusted to pH 5.6 with KOH. Solution B contained the same concentration of TBA and methanol as solution A but lacked the phosphate component (B = 0.7 g/l TBA-Br in 7% methanol). The samples were diluted in water and mixed with a 10× loading buffer prior to analysis as described below, and the HPLC was run isocratically at 1 ml/min with 11% buffer A for the separation of the dNMPs from the corresponding deoxyribonucleosides. The retention times of the dNMPs were determined with a standard sample prior to setting up the HPLC program. The standard samples could be run completely isocratically, whereas assay samples needed an elution step to remove ADP and ATP. For samples with the natural deoxyribonucleoside substrates, the last peak is deoxyadenosine monophosphate (dAMP) at ∼20 min, and the elution is set at 2 min after the dAMP peak. The percentage of solution A is then quickly raised to 90% for an isocratic elution of ATP over 10 min, before quickly returning to initial conditions and equilibrated for 10 min before loading the next sample. The peaks were analyzed by ultraviolet (UV) detection at 270 nm and quantified by comparing the peak heights with a known standard. For testing deoxyadenosine and deoxyadenosine analogs in the enzyme assay, solution A and B were modified by using 5.8% (*v/v*) acetonitrile instead of methanol (i. e. A = 5.8% acetonitrile, 0.7 g/l TBA-Br and 23 g/l KH_2_PO_4_ adjusted to pH 5.6; B = 5.8% acetonitrile and 0.7 g/l TBA-Br). We then used a detection wavelength of 260 nm. Several variants of the HPLC protocol with different percentage of solution A were developed based on previously established principles for nucleotide analysis on the column (26) to be able to separate the products from ATP, ADP, the corresponding substrate and other peaks present in the sample (Supplementary Table S1). Common to all protocols was that the elution of the peaks of interest was performed isocratically, but in some cases we needed an additional elution step with 90% A as described above. Briefly, the HPLC conditions can be categorized into three groups (see Supplementary Table S1 for exact conditions and retention times of the first two groups):

- 75% A (and variants of it): for dAK assays with deoxyadenosine (200 μM assay), F-Ara-A (200 μM assay), F-dAdo, cladribine and clofarabine.
- Low A content (7% or 20%): suitable for dAK assays with Ara-A, deoxyadenosine, FANA-A and F-Ara-A. These protocols require a subsequent switch to 90% A as described above to remove ATP and other late-eluting compounds.
- Low A content (30%), but with the acetonitrile concentration in solution A and B raised to 10% for assays with 2-iodo-deoxyadenosine to compensate for its higher hydrophobicity than the other substrates. The retention times (min) were then: 4.71 (ADP), 6.11 (I-dAdo), 11.04 (ATP) and 16.17 (I-dAMP).

As mentioned above, the samples were generally mixed with water (depending on the dilution factor desired) and 10× loading buffer (one-tenth of the final volume). However, the phosphate content in the 10× loading buffer was varied depending on the percentage of solution A in the mobile phase. Generally, 1 ml buffer 10× loading buffer was prepared by mixing X μl 115 mg/ml KH_2_PO_4_ (adjusted to pH 5.6 with KOH), 500-X μl water and 500 μl 14 mg/ml TBA-Br. The value of X is then adjusted to give a final concentration of KH_2_PO_4_ that is below the corresponding concentration in the mobile phase, whereas the final TBA-Br concentration becomes the same as in the mobile phase. The X values used were the following: X = 100 (protocol with 7% A), X = 200 (protocol with 20% A) and X = 500 (remaining protocols). The loading buffer did not contain any organic solvent and was usable for both the methanol- and acetonitrile-containing mobile phases.

### Determination of dAMP, deoxyadenosine diphosphate (dADP), and dATP present in the dAK preparation

The procedure was similar as described previously for the analysis of nucleotides from cell extracts (26). Briefly, 200 μl 1.15 mg/ml dAK protein was precipitated with 200 μl 10% (*w/v*) trichloroacetic acid and centrifuged for 1 min at 16 000 × *g*. The supernatant was subsequently extracted with 576 μl of a 1:0.28 (*v/v*) mixture of Freon (1,1,2-trifluoro-2,2,1-trichloroethane) and N,N,N-trioctylamine for the removal of trichloroacetic acid. The upper phase was saved and diluted tenfold in water and 10× loading buffer before nucleotide quantification as described in the HPLC analysis section. A volume reduction from 400 μl to 388.6 μl by the removal of 5% TCA after Freon-trioctylamine extraction was taken into account in the calculations of nucleotide content per polypeptide (26).

### *Giardia intestinalis* cultivation and EC_50_ determinations

EC_50_ determinations were performed on the *G. intestinalis* WB cells (parent strain), as well as metronidazole-resistant strains (M1 and M2) and resistant revertant cells (M1-NR) (27). The parasites were cultured in TY-I-S medium as described before (28), with the metronidazole-resistant cells grown in the presence of metronidazole, except for the last passage (24 h) prior to the start of the experiment. For EC_50_ determinations, the cells were distributed on microtiter plates, grown in the presence of various concentrations of drugs over 72 h, incubated with CellTiter-Glo reagent (Promega), counted on an Infinite M200 Pro (Tecan Group, Ltd) and analyzed by the GraphPad Prism 10.1.0 software as describe before (21). The data were fitted to log Inhibitor versus Response curves, and standard errors were determined from the variation of 3–4 independent experiments.

### *Giardia intestinalis* cyst formation

The effect of cladribine on cyst formation is unpublished data from a previous study of the effect of azidothymidine on the encystation process (21). The two negative controls - Control and DMSO (dimethyl sulfoxide) - and the positive control (metronidazole) shown here for comparison are therefore identical with the previous publication, whereas the data on cladribine is new (Supplementary Figures S3 and S4). Cells were resuspended to a density of ∼2.5 × 10^5^ cells and allowed to attach for 3 h in 3 ml Nunc cell culture tubes #156 758 (Thermo Fisher Scientific, Waltham, MA, USA) before exchanging the medium into encystation medium. The tested drugs were added at a final concentration of 10× the determined EC_50_ value (11 μM cladribine) in the beginning of the process (0 h) or at certain time points during the encystation process (4, 8, 12 or 20 h) and the experiment was terminated at 28 h. The drug was solubilized in DMSO prior to use (total volume of 10 μl added to the cells). The negative control was handled in a similar manner and the tubes were opened, closed, and inverted but with nothing added (or 10 μl DMSO in the second control). At 28 h, the cells were centrifuged (800 × *g*), washed twice with sterile H_2_O, resuspended and incubated for 5 days with H_2_O to make the cells permeable to dyes. The cells were finally stained with fluorescein diacetate (FDA) to measure if they were alive and with propidium iodide (PI) to measure DNA content as described before (21,29). Completed cysts are permeable only to FDA, whereas cells caught in the middle of the encystation process are FDA negative but permeable to PI.

### Crystallography, structure solution, and analysis

A solution of 10 mg/ml *G. intestinalis* dAK in a buffer containing 20 mM Tris-HCl (pH = 7.5) and 150 mM NaCl was used to set up sitting-drop vapor diffusion crystallization experiments. Crystals grew in the preformulated Morpheus (30) H3 condition [0.1 M amino acids, 0.1 M Buffer 1 (pH = 6.5), 30% GOL_P4K] within 1–3 weeks. The obtained crystals were cryo-protected by short immersion in a fresh drop of crystallization condition and flash-frozen in liquid nitrogen. Data were collected at the MAXIV BioMAX beamline (31–33), processed with XDS (34) and scaled to 2.1 Å using aimless (35) within the CCP4 suite (36). A total of 5% of total reflections were set aside as an *R*_free_ test set. The crystals belonged to space group P3221 with two molecules per asymmetric unit (ASU), a solvent content of 69.3% and a Matthews coefficient of 4.00. All subsequent data processing was performed within the PHENIX suite (37). The structure was solved with Phaser (38), using a processed AlphaFold-predicted *G. intestinalis* dAK structure (39) as a search model. The structure was fully refined, and the model built using iterative cycles of phenix.refine (40) and COOT (41), respectively. Ligand topology was generated using eLBOW (42). Polder maps were calculated with phenix.polder (43). Data collection and refinement statistics can be found in the supplementary material (Supplementary Table S2). Dimer–dimer interactions were predicted using the PISA server (44).

### Molecular graphics and visualization

Figures of protein structures and electron densities were generated using ChimeraX (45). Ligand topology was generated using LigPlot+ (46).

### Cryo-EM sample preparation

A total of 3 μl of dAK (1 mg/ml) was applied to QUANTIFOIL Cu R300 1.2/1.3 (Electron Microscopy Sciences, catalog no. Q3100CR1.3) that had been glow discharged using a PELCO easiGlow device (Ted Pella Inc.) at 15 mA for 30 s. The sample was applied by transferring 3 μl of sample onto the film-covered side of the grid, blotted and plunge-frozen in liquid ethane, using a Vitrobot plunge freezer (Thermo Fisher Scientific), with the following settings: 4°C, 100% humidity, a blot force of − 5 and a blotting time of 5 s.

### Cryo-EM data collection

Data were collected at an FEI Titan Krios G2 transmission electron microscope (Thermo Fisher Scientific) operated at 300 kV. The objective aperture was 100 μm. The same microscope was used for both data collections, equipped with a Gatan BioQuantum energy filter coupled to a K2 Summit direct detector (Gatan, Inc) (data collection 1), and a Selectris energy filter coupled to a Falcon 4i direct detector (Thermo Fisher Scientific) (data collection 2), respectively. Coma-free alignment was performed with AutoCtf/Sherpa for data collection 1 and EPU for data collection 2. Data were acquired in parallel illumination mode using EPU (Thermo Fisher Scientific software) at a nominal magnification of 215 000× (specimen pixel size 0.63 Å/px for K2, and 0.58 Å/px for Falcon 4i). Exposures were recorded as dose-fractionated movies in TIFF and EER format, for data collections 1 and 2, respectively. Data collection 1 resulted in 2534 movies, whereas data collection 2 resulted in 3420 movies. Complete data collection statistics are shown in Supplementary Table S3.

### Cryo-EM data processing

The processing of the two data collections is outlined in Supplementary Figure S5. For data collection 2, movies were converted from EER to TIFF using IMOD prior to processing (47). Both datasets were processed using RELION (RELION 3.1 (48) for Data Collection 1 and RELION 4 (49) for Data Collection 2). The Beam-induced motion was corrected using RELION’s MotionCor2 implementation (50) and the per-micrograph contrast transfer function (CTF) estimated using GCTF (51) and CTFFIND4 (52) for Data Collection 1 and 2, respectively. Particles were picked using crYOLO (53) and subjected to reference-free 2D classification. Extensive attempts were made at 3D classification in RELION, but no distinct classes with superior 3D appearance to the consensus were obtained. Therefore, we performed 3D refinement using all particles from the selected 2D classes. One class from the 3D classification, low-pass filtered to 20 Å, was used as a reference model. After refinement, a soft-edge mask was created using RELION, and the map was post-processed. Data collection 1 and 2 generated qualitatively similar results. Both volumes were strongly affected by preferred particle orientations, resulting in anisotropic resolution (shown for data collection 2 in Supplementary Figure S6). Due to the slightly different pixel sizes and different detector properties, the data sets were not combined. Data collection 1 generated slightly sharper-looking 2D class averages and was therefore preferred when showing images, whereas data collection 2 generated a 3D map that appeared less deformed by anisotropic resolution, and this map was thus used for the structure determination and for deposition at EMDB. The resolution of the unmasked and masked maps from data collection 2 was calculated using the Gold-Standard Fourier shell correlation (FSC threshold, 0.143) to be 6.3 Å and 4.8 Å, respectively (Supplementary Figure S5). The application of the internal D2 symmetry found in the crystal structure did not significantly improve the map appearance, and only the unsymmetrized C1 map was used. The map was viewed and analyzed using ChimeraX, which was also used to manually fit the crystallographic tetramer to the map (54). To construct 2D projections of the crystal structure, the PDB of the crystal structure was first converted to mrc and low pass-filtered to 7 Å followed by using e2project3d (55) to get 2D projections.

### Size exclusion chromatography

Proteins were analyzed on a Superdex 200 10/300 GL column from GE healthcare (Chicago, IL, USA) equilibrated using a mobile phase containing 150 mM KCl and 50 mM Tris-HCl (pH = 7.6) run at 0.4 ml/min. The proteins were diluted with mobile phase into a concentration of 0.1 mg/ml prior to use, loaded onto the column using a 100 μl sample loop, and measured by a UV detector set at 280 nm. The molecular mass of the protein was assessed by comparing the retention time to a standard curve obtained from a mixture of ovalbumin (45 kDa), transferrin (78 kDa), IgG (150 kDa) and ferritin (440 kDa).

### Mass photometry

The oligomeric state of dAK was analyzed on a Refeyn 2MP mass photometer (Refeyn Ltd, Oxford, UK). Prior to analysis, *G. intestinalis* dAK was mixed with PBS into a final protein concentration of 50 nM. For the calibration of the instrument, NativeMark Unstained Protein Standard (Thermo Fisher Scientific) was used.

## Results

### Sequence analysis of *G. intestinalis* dAK reveals N-terminal and C-terminal extensions

An alignment of the *G. intestinalis* dAK (ORF GL50803-17451 in assemblage A isolate WB) with several bacterial and eukaryotic non-TK1-like dNKs showed that it has longer N- and C-terminal tails than most species (Supplementary Figure S7). It was especially uncommon to have extensions in both directions. The amino acid sequence of the *G. intestinalis* dAK has 38% identity to the second *G. intestinalis* non-TK1-like dNK and 30–34% identity to dAKs from *M. mycoides*, *B. cereus* and *L. acidophilus*, indicating a similarity to bacterial non-TK1-like dNKs (56). For comparison, the corresponding identity to the metazoan dNKs included in the sequence alignment was only 23–26%. Conserved active site residues in the other species were present in the *G. intestinalis* dAK as well (Supplementary Figure S7), but there are also some non-conserved residues in contact with the substrate.

**Figure 2.**
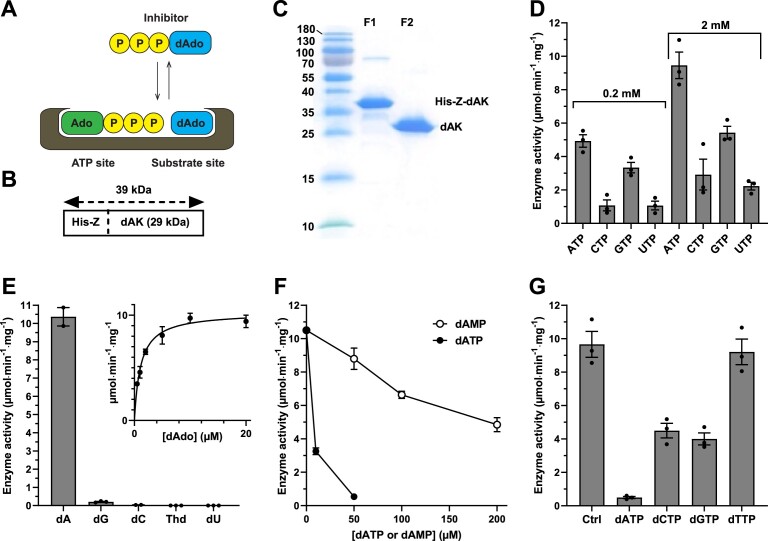
*Giardia intestinalis* dAK purification and characterization. (**A**) Inhibition mechanism of dNKs (dAK in the figure) by the triphosphate form of the substrate. (**B**) *Giardia intestinalis* dAK was expressed as fusion construct with a His-tagged Z protein as a fusion partner. (**C**) Sodium dodecyl sulfate–polyacrylamide gel electrophoresis showing the purity of the protein after nickel agarose (F1), and after fusion partner removal and re-purification (F2). (**D**) Enzyme activity with different phosphate donors present at a concentration of 0.2 mM (left) or 2 mM (right). (**E**) Enzyme activity with all indicated substrates present simultaneously at a concentration of 200 μM each in the assay. (E-inset) Enzyme kinetic analysis with deoxyadenosine as substrate. (**F**) Enzyme inhibition with dATP and dAMP. (**G**) Effect of different dNTPs on enzyme activity. Standard enzyme assay conditions were used in the experiments and unless otherwise stated, the substrate was 200 μM deoxyadenosine and the phosphate donor 2 mM ATP. Graphs D-G represent the average of three independent experiments with standard errors indicated.

In Supplementary Figure S7, residues important for binding the deoxyribonucleoside substrate as well as structural elements important for ATP binding are highlighted. All dNKs have a similar regulatory mechanism with the triphosphate form of the substrate acting as an inhibitor (19). This is exemplified by dATP inhibition of dAK in Figure 2A, where it is shown that dATP interferes with both the binding of the deoxyadenosine substrate as well as with the binding of the phosphate groups of ATP coming from the opposite direction. The binding of dNTPs is therefore dependent on deoxyribonucleoside-interacting residues as well as the P-loop for binding the phosphate groups (Supplementary Figure S7). In addition, the LID domain can sometimes also be involved in the phosphate interaction depending on the type of dNTP and dNK (57). The binding of the nucleoside part of ATP is only mediated by a few backbone interactions and therefore not indicated in the figure.

### *Giardia intestinalis* dAK shows a preference for deoxyadenosine as a substrate and is inhibited by dATP

The *G. intestinalis* dAK gene was cloned into a pET-Z vector for expression in *E. coli* as a fusion construct with a removable N-terminally His_6_-tagged Z protein followed by a TEV protease cleavage site (Figure 2B). The expressed protein was purified by Nickel-NTA agarose chromatography, cleaved with TEV protease, and repurified with Nickel-NTA agarose to collect pure untagged dAK in the flow-through (Figure 2C). Based on initial enzyme activity studies, it was confirmed that the assay did not have any specific requirements regarding K^+^, Na^+^, NH_4_^+^, acetate or reducing agents, and that it was linear with respect to time (Supplementary Figure S1). ATP was the best phosphate donor and deoxyadenosine the best substrate (Figure 2D and E). A kinetic analysis with deoxyadenosine as substrate showed that there was no obvious sign of cooperativity and that the enzyme had K_M_ and V_max_ values of 1.12 ± 0.37 μM and 10.3 ± 1.0 μmol·min^−1^·mg^−1^, respectively (Figure 2E, inset). It has thus a nearly 40-fold higher catalytic efficiency (k_cat_/K_M_) for deoxyadenosine than *M. mycoides* dAK (58).

*Giardia intestinalis* dAK was inhibited by its immediate product, dAMP, and even more efficiently by its far-end product, dATP (Figure 2F). Further analysis showed that dATP affected both K_M_ and V_max_ when deoxyadenosine was the varied substrate, whereas it was a purely competitive inhibitor with respect to ATP (Supplementary Figure S8). From the ATP data, it was possible to calculate a K_i_ value of 0.84 ± 0.22 μM for dATP and a K_M_ value of 350 ± 50 μM for ATP. A previous study on *L. acidophilus* dNKs demonstrated that the effect of dNTPs on K_M_ and V_max_ gives information about if the reaction is ordered or random (59). The profile of the *G. intestinalis* dAK inhibition, which was purely competitive with respect to ATP (i.e. strictly affecting K_M_) but also affecting k_cat_ with deoxyadenosine is indicative of that the reaction is ordered with ATP binding first. In random order reactions, the dNTP is competitive with respect to both ATP and the deoxyribonucleoside (59). The ordered mechanism of the *G. intestinalis* dAK indicates that the phosphate groups are important for binding to the free enzyme, giving an explanation why dAMP with only one phosphate group is a weaker inhibitor in comparison to dATP.

Other dNTPs could also inhibit *G. intestinalis* dAK activity with a potency that mirrored the efficiencies of the corresponding deoxyribonucleosides shown in Figure 2E and Table 1; dATP was the strongest inhibitor whereas a much weaker effect was obtained with dGTP and dCTP (Figure 2G). As expected from this rationale, dTTP did not inhibit the reaction.

**Table 1.** Enzyme kinetics of *G. intestinalis* dAK with different substrates

Substrate	K_M_ (μM)	V_max_ (μmol·min^−1^·mg^−1^)	k_cat_ (s^−1^)	k_cat_/K_M_ (M^−1^·s^−1^)
dAdo	1.12 ± 0.37	10.3 ± 1.0	4.96	4.43 × 10^6^
dGuo	476 ± 133	4.87 ± 0.49	2.36	4.94 × 10^3^
dIno	701 ± 83	9.49 ± 1.14	4.59	6.54 × 10^3^
dCyd	2090 ± 380	12.1 ± 1.3	5.87	2.81 × 10^3^
dThd		<0.25*		
dUrd		<0.1*		
Ado	27.1 ± 1.9	2.58 ± 0.09	1.25	4.61 × 10^4^
Cladribine (Cl-dAdo)	0.51 ± 0.08	5.78 ± 1.16	2.79	5.48 × 10^6^
I-dAdo	4.78 ± 0.17	13.7 ± 0.8	6.63	1.39 × 10^6^
Ara-A	6.70 ± 0.07	6.60 ± 0.06	3.19	4.76 × 10^5^
F-Ara-A	8.40 ± 0.60	7.20 ± 0.82	3.48	4.14 × 10^5^
FANA-A	2.66 ± 0.11	7.64 ± 0.64	3.69	1.39 × 10^6^
Clofarabine (Cl-FANA-A)	6.23 ± 1.33	7.26 ± 1.32	3.51	5.63 × 10^5^
**Truncated dAK (dAK-ΔNΔC**)				
dAdo	53.8 ± 3.10	13.0 ± 1.9	5.22	9.69 × 10^4^
dCyd	31 600 ± 6600	11.0 ± 1.7	4.40	139

*Numbers indicate the detection limit in activity assays with 2 mM substrate.

The truncated dAK is described in the mutational analysis section. The values indicate the average with standard errors from three independent experiments. The k_cat_ value is shown as activity per polypeptide.

Ara-A, adenine arabinoside; FANA-A, 2′-fluoro-β-d-arabinosyl adenine (additional F-, Cl- and I-labels indicate modifications in the 2 position of the adenine moiety).

### *Giardia intestinalis* dAK has a high catalytic efficiency with deoxyadenosine and analogs modified at position 2 and 2′

**Figure 3.**
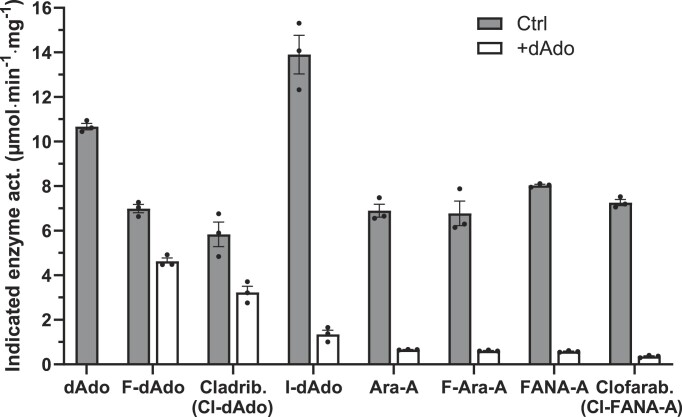
*Giardia intestinalis* dAK is active with deoxyadenosine analogs modified at the 2 and 2′positions. The activity assays were performed with 200 μM analog in the absence or presence of 200 μM deoxyadenosine to get information about both activity and affinity. The recorded enzyme activities are for the substrates indicated on the x-axis (competing dAdo activity was not measured in the white bar experiments). The results are based on three independent experiments with standard errors indicated.

Enzyme kinetic parameters of the *G. intestinalis* dAK activity were determined with natural deoxyribonucleosides as well as deoxyadenosine analogs (Table 1). Among the natural deoxyribonucleosides, deoxyadenosine stands out by its low K_M_ value whereas the differences in V_max_ were much less pronounced. Substrate selection is thus primarily affinity-based with deoxyadenosine being strongly preferred over the others. However, it cannot be excluded that the low-affinity substrates may be relevant as well in the cell where other factors such as transport efficiency and further metabolism into the triphosphate form can play important roles. Another finding was that the catalytic efficiency (k_cat_/K_M_) with adenosine was 100-fold lower than with deoxyadenosine, suggesting that the enzyme does not use ribonucleosides efficiently. This is a common feature of dNKs (19). Table 1 also shows the efficiency of different deoxyadenosine analogs as substrates. To validate the results with the deoxyadenosine analogs shown in Table 1, we also made an alternative assay where each analog was tested at 200 μM concentration with and without 200 μM deoxyadenosine as competitor (Figure 3). The substrate concentration was chosen to be well above the K_M_ for all substrates. The results in Figure 3 matched well with the conclusions in Table 1. When tested alone, the analogs gave activities that were very similar to the V_max_ values and in the competition experiments, deoxyadenosine could efficiently compete out the high-K_M_ substrates (>90% inhibition) but had <50% effect on the 2-chlorinated/fluorinated versions of deoxyadenosine (45% effect with cladribine and 33% with F-dAdo). Both analogs are therefore likely to be low K_M_-substrates although it was only cladribine that could be measured in Table 1 (submicromolar K_M_-measurements requires radiolabeled substrates). However, deoxyadenosine itself also has a high affinity and the beneficial effect of 2- chlorination/fluorination was modest and not comparable to the 20-fold improvement reported for the human dCK that is responsible for deoxyadenosine phosphorylation in the cytosol (60). Nevertheless, *G. intestinalis* dAK is generally a much more active enzyme than the human dCK and the catalytic efficiency is > 50-fold higher with cladribine and > 1000-fold higher with deoxyadenosine. 2-Iodo-deoxyadenosine was also tested as substrate of the *G. intestinalis* dAK and was found to have the highest k_cat_ value of all tested substrates, but the catalytic efficiency (k_cat_/K_M_) was still lower than that of cladribine because of their K_M_ differences (Table 1).

A general trend for the deoxyadenosine analogs in Table 1 is that the K_M_ value for deoxyadenosine is lower than the corresponding arabinosyl- and 2′-F-substituted arabinosyl derivatives (Ara-A and FANA-A), which was in line with the conclusions from the competition assay. Nevertheless, all deoxyadenosine analogs tested had catalytic efficiencies within approximately one order of magnitude and can thus be considered good substrates. The effect of 2-halogenation varied but was never as prominent as for the mammalian dCK and led to reduced catalytic efficiency in the case of clofarabine (Table 1).

### Deoxyadenosine analogs inhibit *G. intestinalis* proliferation and encystation

A growth inhibition assay with different deoxyadenosine analogs showed that they inhibited the *G. intestinalis* parasites with EC_50_ values that mirrored the substrate specificity of dAK. The EC_50_ values of the analogs on the WB strain were in the range from 1–7 μM (Table 2), and comparable to metronidazole which gives an EC_50_ value of 2.7 μM on the same strain under these conditions (21). Among the analogs tested here, cladribine was the most active one, whereas a higher selectivity as compared to the mammalian reference cell line was obtained with Ara-A and F-Ara-A (Table 2 and Supplementary Figure S9). Note that a main contributor to the selectivity of Ara-A is that the drug is deaminated by the mammalian cells but not by the parasites. Accordingly, our previous analysis shows an EC_50_ value of 1.8 μM on Balb/3T3 fibroblasts (and > 10 μM on human WS1 fibroblasts) when the drug is combined with 1 μM EHNA to inhibit mammalian adenosine deaminase (61). In the case of cladribine and F-Ara-A, they are deamination resistant and the results with these analogs are not dependent on the presence of adenosine deaminase inhibitors. All analogs tested were fully active on metronidazole-resistant *G. intestinalis* strains as well, which is important for their potential as drugs against giardiasis. Cladribine was also verified to nearly completely inhibit the ability of the parasites to form cysts if added in the beginning of the encystation process and reduced the number of mature cysts with a 16N genome if added later (Supplementary Figure S3 and S4). A lower number of 16N cells is indicative of inhibited DNA replication during encystation. In conclusion, the experiments on cells provide a proof of principle that deoxyadenosine analogs can inhibit the pathogen in the low micromolar range and are efficient regardless of metronidazole-resistance.

**Table 2.** EC_50_ values showing the inhibition of *G. intestinalis* and mammalian fibroblast (Balb/3T3) proliferation

	**WB EC_50_ (μM**)	**M1 EC_50_ (μM**)	**M2 EC_50_ (μM**)	**M1NR EC_50_ (μM**)	**Balb/3T3 EC_50_ (μM**)	**Selective index**
Cladribine	1.1 ± 0.18	1.0 ± 0.07	1.0 ± 0.05	0.8 ± 0.06	0.78 ± 0.25	0.71
F-Ara-A	7.0 ± 2.7	4.0 ± 1.08	2.3 ± 0.38	3.5 ± 0.5	60 ± 15	8.6
Ara-A	5.5 ± 0.5	5.8 ± 0.95	3.0 ± 0.25	3.8 ± 0.41	230 ± 140*	42

*Variable results on mammalian cells (due to the absence of an adenosine deaminase inhibitor).

The experiments were performed with wild-type *G. intestinalis* cells (WB strain), metronidazole-resistant cells (M1 and M2) and resistance-revertant cells (M1NR). The values represent the results from three independent experiments with standard errors indicated.

### Structure determination of *G. intestinalis* dAK reveals a homotetramer formed by a combination of novel and canonical dimerization interfaces

**Figure 4.**
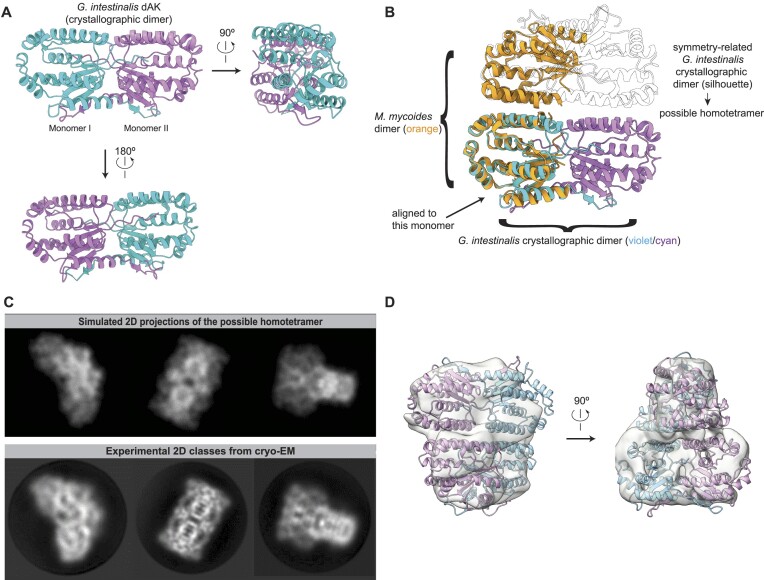
Structure determination of *G. intestinalis* dAK reveals a homotetramer formed by a combination of novel and canonical dimerization interfaces. (**A**) Three different views of the crystallographic dimer of *G. intestinalis* dAK at the indicated rotations. The two subunits are shown in violet and cyan. (**B**) The crystallographic dimer of *G. intestinalis* (cyan/violet) superimposed on the structure of *M. mycoides* dAK dimer (orange, PDBID: 2JAQ). In the crystal of *G. intestinalis* dAK, a symmetry-related dimer (shown as a silhouette) interacts with the crystallographic dimer through a similar interface as that seen in the *M. mycoides* dimer. This indicates a possible tetrameric assembly of the *G. intestinalis* dAK (cyan/violet/silhouette). (**C**) The top row shows computational simulations (reprojections at 7 Å resolution) of cryo-EM 2D class averages that the possible homotetramer would lead to. The lower row shows corresponding experimental 2D class averages from cryo-EM of *G. intestinalis* dAK. (**D**) The homotetramer model fitted in the anisotropic 3D density map obtained by cryo-EM (C1 symmetry, nominal resolution 4.8 Å). Panels (C) and (D) were prepared from data collection 1 and 2, respectively.

To structurally characterize the active site and investigate the substrate preference of *G. intestinalis* dAK, we solved the structure of full-length *G. intestinalis* dAK to 2.1 Å by macromolecular X-ray crystallography (Figure 4A and Supplementary Table S2). The ASU contained two copies of *G. intestinalis* dAK, of which each subunit showed a similar fold to the homologous structure of *M. mycoides* dAK, as well as human dGK and dCK (Supplementary Figure S10) (57,62,63). However, when we superimposed the *G. intestinalis* dAK dimer contained in the ASU with the previously reported *M. mycoides* dAK dimer, we noticed that *G. intestinalis* dAK dimerizes through a different, not previously reported, interface (Figure 4B, comparison of violet/cyan and orange dimers). In the dAK crystal, a neighboring, symmetry-related dAK dimer was located such that it made similar protein-protein interactions with the ASU dimer as seen between monomers in the *M. mycoides* dimer (Figure 4B, in silhouettes). We term the dimerization interfaces found in the ASU (Figure 4A) and the one similar to the *M. mycoides* (Figure 4B) the ‘novel’ and ‘canonical’ interfaces, respectively. To ascertain whether the novel and canonical interfaces were artefacts of crystal packing or reflected the true state of the enzyme in solution, we studied the dAK structure using cryo-electron microscopy (cryo-EM) single particle analysis (Supplementary Figure S5). If both the novel and canonical interfaces also form in solution, dAK would form the homotetramer indicated in Figure 4B. The experimental 2D class averages obtained by cryo-EM closely resembled computer-generated 2D re-projections of the putative homotetramer from the crystal structure (Figure 4C). Due to strong preferred orientations, it was not possible to get an isotropic, high-resolution 3D structure of dAK using cryo-EM. The resulting 3D map had a nominal resolution of 4.8 Å, but a clearly anisotropic appearance probably reflecting lower resolution in the poorly resolved directions (Supplementary Figure S6). However, the 3D map was sufficient to perform an approximate fitting of the crystal structure tetramer, whose size and shape fitted the cryo-EM map well at the attained resolution (Figure 4D). Taken together, the convergence of X-ray crystallography and cryo-EM revealed that *G. intestinalis* dAK is a homotetramer, and that tetramerization is mediated by a combination of a novel dimerization interface and the canonical one also found in *M. mycoides* dAK.

### Co-purified deoxyadenosine mono- and diphosphate ligands bound to *G. intestinalis* dAK allow for the modelling of substrate analogs

After crystallographic structure solution by molecular replacement, we identified unbiased *F_o_-F_c_* density that corresponds to ligands bound in the active sites of the crystallographic dimer. Interestingly, no substrates or analogs had been added during protein purification or during crystallization, suggesting that the bound ligands were present already in the cell extract and exhibit a high binding affinity for dAK. The ligand density was well enough resolved to identify shapes corresponding to an adenine and a deoxyribose sugar. However, the number of phosphate groups differed in the two subunits: whereas a dADP, with a partial occupancy of 0.5 for the β-phosphate, could be modelled into active site I, the density for active site II unambiguously allowed for the placement of a dAMP (Supplementary Figure S11). To increase our confidence that dADP was correctly placed within active site I, we calculated a polder map for dADP to test whether the β-phosphate density is correctly attributed or if it rather belongs to the bulk solvent. The resulting polder map and statistics support the placement of dADP in active site I with correlation coefficients of CC_1,2_= 0.7627, CC_1,3_= 0.9424 and CC_2,3_= 0.7423 suggesting that the density does belong to dADP as CC_1,3_ > CC_1,2_ and CC_1,3_ > CC_2,3_ (Supplementary Figure S11). HPLC analysis of the dAK preparation showed that the protein contains 0.40 dAMP and 0.35 dATP per polypeptide, and subsequent analysis showed that dATP could slowly be converted to dADP by the enzyme preparation over a matter of days at room temperature, which is consistent with a dATP-to-dADP conversion during crystallization (Supplementary Figure S2). The dATP-dephosphorylating activity was several orders of magnitude lower than the regular dAK activity (to phosphorylate deoxyadenosine) and is possibly catalyzed by other enzymes present as minor impurities in the protein preparation. The overall conclusion is that monomer I is a mixed site containing either dAMP or dADP/dATP and monomer II is dominated by dAMP. In other aspects, the two subunits are very similar to each other and the active sites are virtually identical except for some subtle changes to accommodate the β-phosphate in monomer I. Note that it is not possible to say from the crystal structure data if the two subunit conformations are stable in solution where nucleotide binding is an equilibrium process and the protein is more flexible. An alternative could be that the subunits rapidly switch between the two conformational states depending on which nucleotide is bound at any given moment.

**Figure 5.**
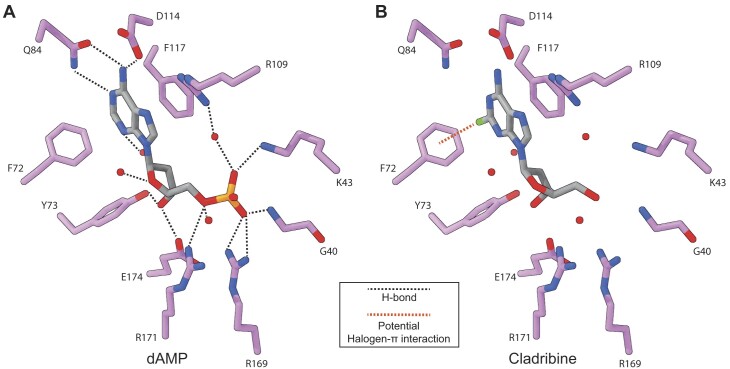
The active site of *G. intestinalis* dAK reveals an extensive hydrogen bond pattern. (**A**) Stick representation of dAMP (gray sticks) bound by active site residues (magenta sticks) of *G. intestinalis* dAK. Water molecules are shown as red spheres and hydrogen bonds as black dotted lines. (**B**) Representation of cladribine (gray sticks) modelled into the active site of *G. intestinalis* dAK, using the adenine base as reference. A potential, additional halogen···π-interaction is shown as an orange dotted line.

Most of the active site residues of *G. intestinalis* dAK are conserved to the residues found in *M. mycoides* dAK as indicated in Supplementary Figure S7, although there are a few differences when looking at how they interact with the adenine moiety. In *G. intestinalis* dAK, the adenine base is held in place by face-to-edge (F72) and face-to-face (F117) π-stacking, and hydrogen bonds between the adenine nitrogen N1, N3 and N10 and Q84, an H_2_O molecule and Q84 and D114, respectively (Figure 5A). For comparison, the *M. mycoides* dAK has an additional hydrogen bond to the adenine moiety (R61 binds N7), and it has a tyrosine instead of F72. The arginine is present in *G. intestinalis* dAK as well (R91), but in our structure it is too far away to interact with the adenine base although it cannot be excluded that it can rotate into a more favorable position when the protein is in solution. The other adenine interacting residues are conserved including F117 (F86 in *M. mycoides*), Q84 (Q54 in *M. mycoides*) and D114 (D83 in *M. mycoides*). The deoxyribose oxygen present in the ring forms hydrogen bonds with an H_2_O molecule and the 3′-hydroxy group forms hydrogen bonds with E174 and Y73 (Figure 5A), which are conserved in all aligned species in Supplementary Figure S7. Finally, the α-phosphate forms hydrogen bonds with G40, K43, R109 via an H_2_O molecule, and R169. Our biochemical studies indicated that cladribine is a slightly better substrate than deoxyadenosine. By using the adenine base as reference, we therefore modelled cladribine into the active site of *G. intestinalis* dAK (Figure 5B). The modelled cladribine ligand suggested that the 2-Cl group points towards F72, altering the face-to-edge π-stacking to a halogen-π interaction (Figure 5B), which has been shown to be a stronger type of interaction (64,65). This provides a possible explanation for how cladribine and F-dAdo can have a higher affinity than deoxyadenosine. However, the model did not demonstrate a perfectly perpendicular face to edge interaction and the gain in affinity compared to deoxyadenosine was modest (Table 1 and Figure 3), indicating a potential for ligand improvement in drug development. Molecular modelling also gave insights into why adenosine is a poor substrate in comparison to deoxyadenosine (Supplementary Figure S12). The modelling indicated a possible clash between the 2′-hydroxyl group and a nearby tyrosine (Y73).

### The N- and C-termini of dAK form extensive interactions with the neighboring subunit across the novel interface

**Figure 6.**
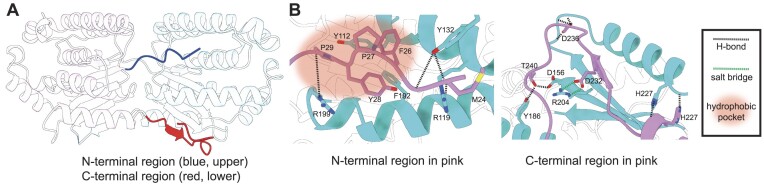
The N- and C-termini of dAK form extensive interactions with the neighboring subunit across the novel interface. (**A**) Illustration of the X-ray crystal structure showing how the N-terminus (blue) and C-terminus (red) of one subunit interacts with a neighboring subunit across the novel interface. The termini of the other subunit makes identical interactions on the back of the shown dimeric subcomplex, which constitutes half of the full homotetramer. (**B**) Detailed representation of the of N- (left) and C-termini (right) interaction pattern with the neighboring monomer. N-terminal interactions in the hydrophobic pocket (MI: monomer 1, MII, monomer 2): F26_MII_, Y28_MII_, P27_MII_, P29_MII_, Y112_MI_, F192_MI_. N-terminal hydrogen bonds: M24_MII_:R119_MI_, M24_MII_:Y132_MI_, G25_MII_:Y132_MI_, P29_MII_:R199_MI_. C-terminal salt bridges: D232_MII_:R204_MI_. C-terminal hydrogen bonds: H227_MI_:H227_MII_, R206_MI_:D236_MII_, A208_MI_:D236_MII_, Y210_MI_:D236_MII_, D156_MI_:T240_MII_, Y186_MI_:T240_MII_. The hydrophobic pocket is shown as a transparent orange oval, hydrogen bonds as black dotted lines, and salt bridges as green dotted lines. Protein chains are shown in cartoon representations and interacting residues as sticks.

In the analysis of the dAK structure, we next turned our attention to the details of the novel interaction interface. In the crystal structure, clear electron density was visible for large parts of the extensions at the termini of the protein. The N- and C-terminal extensions reached across the novel dimerization interface to interact with the neighboring subunit of the tetramer (Figure 6A, half of the tetramer is shown). Due to symmetry, the novel dimerization interface is thus stabilized by identical N-terminal and C-terminal interactions from each subunit with the other subunit, and the full tetrameric assembly contains four each of these interactions. Residues close to the N-terminus (M24_MI_-P29_MI_) of the first monomer (MI) form extensive interactions with the residues of the second monomer (MII) (Y112_MII_, R119_MII_, Y132_MII_, F192_MII_, R199_MII_) (Figure 6B and Supplementary Table S4). Hydrogen bonds are formed between the sidechains and amide backbone oxygen of Y132_MI_:M24_MII_, R119_MI_:M24_MII_, Y132_MI_:G25_MII_ and R199_MI_:P29_MII_ whereas Y112_MI_ and F192_MI_ together with F26_MII_, P27_MII_ and Y28_MII_ form a hydrophobic pocket (Figure 6B). The C-terminus of MII forms an intermolecular β-sheet with the twisted four-stranded β-sheet of MI in addition to a salt bridge between R204_MI_ and D232_MII_, and H-bonds between D156_MI_:T240_MII_, Y186_MI_:T240_MII_, A208_MI_:D236_MII_, Y210_MI_:D236_MII_, R211_MI_:D236_MII_ and H227_MI_:H227_MII_ (Figure 6B). Thus, the extensions of the dAK N- and C-termini make multiple, specific interactions that appear to stabilize the novel dimerization interface that leads to homotetramerization.

### Deletion mutants of *G. intestinalis* dAK show that the N- and C-termini are important for tetramerization and substrate affinity

**Figure 7.**
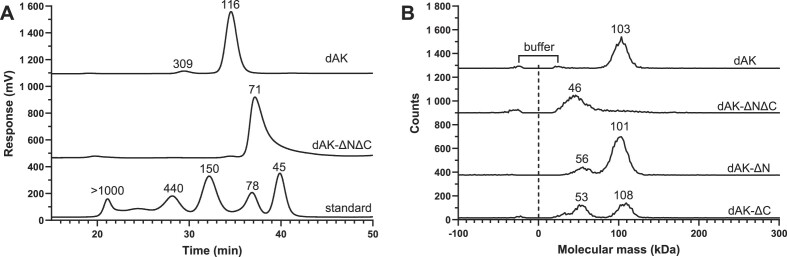
Size exclusion chromatography and mass photometry analysis of full-length and truncated *G. intestinalis* dAK confirm that the N- and C-termini are important for tetramer formation. (**A**) Size exclusion chromatography of full-length dAK, truncated dAK lacking the N- and C termini (dAK-ΔNΔC) and a protein standard. (**B**) Mass photometry experiments of wild-type dAK, dAK-ΔNΔC, dAK-ΔN and dAK-ΔC. The molecular masses of the dAK peaks in panels (A) and (B) were assessed by comparing them to a standard curve of proteins with known molecular masses. In panel (B), the mass assessment was preceded by first fitting the peaks to Gaussian curves by the instrument software. The buffer peak indicated in panel (B) deviates from a typical protein peak by having equally sized positive and negative peaks coming from binding and unbinding events, respectively. The different graphs in panels (A) and (B) are shifted along the ordinate in order to fit many experiments in the same panel.

In order to study the functional relevance of tetramerization, a deletion mutant of dAK was created that lacks residues 1–29 and 233–251 of the N- and C-termini (dAK-ΔNΔC), which were indicated to be important for subunit interaction in the crystal structure. Size exclusion chromatography indicated that the deletion mutant indeed formed a structure considerably smaller than a tetramer. However, the estimated molecular mass of 71 kDa is in between a dimer (45 kDa) and a tetramer (90 kDa) based on comparison with standard proteins (Figure 7A). The peak exhibited considerable tailing indicating that the mass estimation was not reliable and could be the effect of equilibration between different forms or interaction with the chromatography material. In contrast, the wild-type protein gave a symmetrical peak and clearly indicated a tetramer (theoretically 116 kDa). Using mass photometry, it was possible to get clearer results with the deletion mutant. This method requires much lower protein concentrations (typically < 100 nM) than size exclusion chromatography. The wild-type protein still behaved as a tetramer at 65 nM concentration, whereas measurements of the deletion mutant clearly indicated that it is predominantly a dimer at this protein concentration (Figure 7B). The results supported the view that the size exclusion chromatography (run at 4 μM protein) may result in a dimer-tetramer equilibrium that almost completely shifts to dimer at the lower protein concentration used in mass photometry (65 nM). Mass photometry experiments on other deletion mutants lacking either the N- or C-terminus showed that they were able to form tetramers but with a weaker interaction as indicated by a significant proportion of dimers (Figure 7B). Especially the C-terminus was important for tetramer formation. In contrast, the dimers were undetectable in wild-type dAK samples. Enzyme activity assays with the double deletion mutant (dAK-ΔNΔC) showed that the lost ability to readily form tetramers was accompanied by a ∼50 times increased K_M_ value for deoxyadenosine but no major change in the k_cat_ value (Table 1). The results indicate that it is only the substrate affinity and not the catalytic mechanism that is dependent on the ability to form tetramers. Corresponding experiments with deoxycytidine as an example of a structurally different substrate showed a similar trend with the K_M_ value being much more affected than k_cat_. Thus, the reduced deoxyadenosine affinity in the double-deletion mutant seems to be a general loss of substrate affinity rather than a change in specificity.

## Discussion

The ability to live without RNR seems to be a daunting task considering how few organisms have succeeded. There are several challenges to overcome, with the most obvious one being the scarce supply of substrates for salvage synthesis of dNTPs. The concentration of deoxyribonucleosides is generally below the detection limit in most environments and none of the RNR-lacking organisms found so far is free-living. In essence, it should be possible to degrade DNA from dead cells in the surroundings as a source for salvage but given that no free-living organism has succeeded to live entirely on salvage, it is likely to be difficult and it should be remembered that also among pathogens very few have succeeded.

*Giardia intestinalis* seems to be adapted for the problem of scarce sources or deoxyribonucleosides in several ways. The location of the parasites in the duodenum is one important factor. In this part of the gastrointestinal system, the food is processed and thereby available for the parasite and there are also few other organisms that it needs to compete with. Nevertheless, it is still a challenge to compete with the mammalian cells for the absorption of the deoxyribonucleosides and it therefore needs an efficient system for uptake and salvage. In the case of *G. intestinalis* dAK, this has been accomplished by having a > 1000 times higher catalytic efficiency (k_cat_/K_M_) than the human dCK and dGK have for deoxyadenosine (60,66,67). In fact, none of the studied dNKs up to date has such a high catalytic efficiency for deoxyadenosine as the enzyme from *G. intestinalis*. The high substrate affinity of *G. intestinalis* dAK goes hand in hand with the previous studies of the TK of the parasite, which is able to work efficiently at a much lower thymidine concentration than mammalian TK1 (K_M_ values: 0.07 versus 0.5 μM) (21). It is typical that members from the TK1-family have higher affinities than non-TK1-like dNKs, and the K_M_ of the *G. intestinalis* TK needs therefore to have an exceptionally low K_M_ to be able to compete with the mammalian enzyme. The current study indicates that it could be a general trend that *G. intestinalis* has developed high-affinity dNKs to be able to compete with its host although it remains to be discovered which enzyme is responsible for the phosphorylation of deoxycytidine and deoxyguanosine in the parasite. Although dAK has comparable k_cat_ values for these substrates as for its main substrate, the affinity for them is much lower and it is therefore more likely that the second, not yet studied, non-TK1-like dNK in the parasite is responsible. In conclusion, the parasite seems to have developed highly competitive enzymes for deoxyribonucleosides, at least for deoxyadenosine and thymidine. In the case of deoxyadenosine, a K_M_ of 1.1 μM is low enough to be competitive in comparison to the corresponding mammalian enzymes, whereas for thymidine a much lower K_M_ is required (0.07 μM).

A second challenge for *G. intestinalis* is how to regulate the salvage of deoxyribonucleosides. A hint of how important that is comes from a study of an engineered *E. coli* cell line lacking all RNR genes and supplemented with the *M. mycoides* dAK gene to complement its own partial salvage capacity and be able to proliferate in the presence of deoxyribonucleosides in the medium (68). Over time the cell line downregulated deoxyribonucleotide degradation pathways and gradually evolved the ability to live on lower and lower concentrations of deoxyribonucleosides in the medium. However, it came with the cost of a substantially increased mutation rate. This highlights the key role of RNR not only to produce deoxyribonucleotides but also for the regulation of the process to keep the dNTP pools balanced and minimize replication errors. It is not completely clear how *G. intestinalis* dAK is able to balance the dATP pool in comparison to other dNTPs, but one part of the explanation could be its strong feedback inhibition by dATP (K_i_= 0.84 μM). An important aspect of this regulation is the ordered kinetics with dATP primarily competing with the first substrate to bind (ATP). In this way, the inhibitory effect cannot be outcompeted by excessive deoxyadenosine concentrations although the binding sites are overlapping. The most obvious purpose of the regulation is to efficiently turn the enzyme activity off if dATP is not immediately used up for DNA synthesis. However, another advantage of the strong dATP inhibition is to secure that the substrate is spread equally among the cells in the population. With uncontrolled salvage, there is a risk that the first parasites that encounter the substrate will take the major share and leave too little for the others.

The finding of the much higher catalytic efficiency of *G. intestinalis* dAK for deoxyadenosine as compared to the mammalian dNKs is valuable for drug discovery. One way to target the dependency on salvage would be to make inhibitors against dAK, but that requires development of drugs from scratch, and we have instead focused on substrate analogs which after phosphorylation by the enzyme target downstream processes. The potential advantage of using substrate analogs is then both based on the higher substrate affinity of *G. intestinalis* dAK as compared to the mammalian dNKs and on the fact that the parasite cannot become resistant by downregulating dAK without also losing its ability to replicate. Clinically used deoxyadenosine analogs are primarily targeting cancer cells or virus-infected cells. The benefit of using existing drugs is that many of the pharmacological properties are already known, and fewer clinical trials are needed before it can become a final drug against the parasite. The three most used deoxyadenosine analogs against cancer are cladribine, fludarabine and clofarabine, which block cell division by inhibiting RNR and interfering with DNA replication. They are all halogenated at position 2 of the nucleobase to make them stable against deamination by the mammalian adenosine deaminase. The analogs can be given orally as well as intravenously with the latter option giving higher blood concentrations for a given dose, which is generally preferred in anticancer treatment. In the case of *G. intestinalis*, oral treatment is more relevant due to the localization of the parasite in the duodenum. An advantage is then that the drugs will not be diluted in the blood before they can reach the parasites, which hopefully means that lower doses can be used.

*Giardia intestinalis* differs from mammalian cells in several ways of importance for the development of deoxyadenosine analogs. First, the parasite lacks adenosine deaminase and it is therefore less critical that the drug is protected against deamination although some protection may still be needed for the drug to be stable enough to reach the site of infection. Second, *G. intestinalis* also lacks RNR and it is therefore only the direct interference on DNA replication that is relevant for the antigiardial effect. Our experiments demonstrated that the parasite was sensitive to deoxyadenosine analogs that effectively inhibited proliferation as well as encystation. Cladribine (Cl-dAdo) had the highest catalytic efficiency with *G. intestinalis* dAK of the drugs tested, and modeling of the substrate analog into the *G. intestinalis* dAK structure indicated that a halogen… π interaction between F72 in the protein and the chlorine at position 2 in the substrate contributed to the affinity (Figure 5B). However, the 2-chlorination only gives a 2-fold decreased K_M_ value compared to deoxyadenosine and virtually no change in catalytic efficiency (the k_cat_ is lower). The effect is thus modest as compared to the mammalian dCK and dGK where 2-chlorination leads to a 10–25-fold increased catalytic efficiency (60,67). Although this is far from compensating the superior catalytic efficiency of *G. intestinalis* dAK, it should be remembered that the parasites lack one of the drug targets (RNR) and the experiments on cells showed nearly identical EC_50_ values on *G. intestinalis* as compared to mammalian fibroblasts. Better selectivity could instead be obtained with the two drugs F-Ara-A and Ara-A. Similarly to cladribine, F-Ara-A is 2-halogenated but with the advantage of not being as good substrate for mammalian dCK and dGK (67,69). Ara-A, which is an even worse substrate of dCK and dGK and is mainly phosphorylated by adenosine kinase in mammalian cells, could perhaps be the most interesting option of the two drugs against giardiasis. Clinically it has been used as a topically applied drug against herpes simplex virus and is generally regarded non-toxic if ingested due to the rapid deamination in the body (https://www.rxlist.com). Ara-A (as well as F-Ara-A) is generally given in a phosphorylated form (Ara-AMP) to increase its solubility and needs to be dephosphorylated in the body before it can be taken up by cells. An advantage for the treatment of giardiasis is that the phosphorylated form of the drug is protected against adenosine deaminase, which increases the chances that it can reach the site of the parasites intact. Once there, Ara-A will be liberated from Ara-AMP by intestinal dephosphorylating activities. If needed for stability, the drug can also be combined with an adenosine deaminase inhibitor. However, then it is important to be aware that conventional adenosine deaminase inhibitors used as anticancer drugs are optimized for a strong systemic inhibition of the enzyme and associated with side effects. In the case of giardiasis, it is sufficient to inhibit the enzyme in the local intestinal environment for a limited time and then weaker inhibitors can be considered. A wide range of non-toxic substances that inhibits the enzyme has been described, including garlic and other plant extracts (70).

The crystal structure of *G. intestinalis* dAK sheds light into how the enzyme has attained such a high affinity to its main substrate and how it can be exploited for drug discovery. From a drug development viewpoint, more selective nucleoside analogs can be developed by taking advantage of the enzyme structure. The mammalian dCK does for example not use a halogen-π interaction with the 2-chloro atom in clofarabine and the difference between the enzymes means that it may be possible to develop analogs with other 2-modifications that fit better with the *G. intestinalis* dAK than the mammalian enzyme. Especially, it would be valuable to avoid the beneficial effect that the 2-halogen has on binding to the mammalian dCK and dGK.

The structure of the *G. intestinalis* dAK also gave insights into how such a strong affinity for deoxyadenosine has been obtained. This is the first example of a non-TK1-like dNK with a tetramer structure and subsequent mutagenesis analysis showed that the tetramer structure and the affinity are connected. By deleting the N- and C-termini and thereby breaking up the enzyme into dimers, the enzyme lost its high substrate affinity. Notably, the enzyme kinetics revealed no signs of cooperativity (only hyperbolic Michaelis–Menten curves), ruling out that this would be the reason for the enhanced substrate affinity of the tetramer. This means that the enhanced substrate affinity in the tetramers versus dimers is independent on if there is a substrate bound to the neighboring subunits or not. Also, the tetramerization-mediating termini are not directly interacting with the active site of the neighboring subunit. Thus, we cannot conclusively determine the structural reason why tetramerization increases dAK substrate affinity. It is possible that the extended termini, through their interactions with other parts of the protein, lead to subtle conformational changes that transmit through the protein and optimize active site geometry and hence substrate affinity. Notably, the k_cat_ was not changed significantly upon deleting the extended termini, indicating that the enzyme did not lose its ability to catalyze the reaction. Interestingly, the dimeric enzyme lost affinity for both deoxyadenosine and deoxycytidine indicating that this was a general effect and not a change in specificity. The ability to gain substrate affinity by tetramerization could possibly represent an evolutionary shortcut explaining how the ancestor to the parasite became independent of *de novo* dNTP synthesis. For comparison, changes more directly associated to the active site are likely to cause undesired side effects on catalysis or specificity, which must be handled with compensatory mutations. The tetramerization is an elegant way to directly increase the affinity without affecting other parameters and may be a feature also in other dNKs that can explain how organisms can become able to survive solely on salvage for dNTP synthesis.

## Supplementary Material

gkae1073_Supplemental_File

## Data Availability

Coordinates reported in this study have been deposited with the Protein Data Bank with accession code 8PUU. Electron microscopy maps and half-maps have been deposited in the Electron Microscopy Data Bank with the accession code EMD-17948.
