# Supplementary material for: Tetramerization of deoxyadenosine kinase meets the demands of a DNA replication substrate challenge in Giardia intestinalis
Source: Nucleic Acids Res. 2024 Nov 28;52(22):14061–76. doi: 10.1093/nar/gkae1073 (PMC11662666; doi:10.1093/nar/gkae1073)
Supplement: gkae1073_Supplemental_File [file gkae1073_supplemental_file.pdf]

SUPPLEMENTARY MATERIAL

Tetramerization of deoxyadenosine kinase meets the demands of a DNA replication substrate challenge in *Giardia intestinalis*

Farahnaz Ranjbarian[#], Karim Rafie[#], Kasturika Shankar[#], Sascha Krakovka, Staffan G. Svärd, Lars-Anders Carlson* and Anders Hofer*

[#]Shared first authors
*Shared corresponding authors: lars-anders.carlson@umu.se and anders.hofer@umu.se

Table of Contents

Table S1. Effect of HPLC conditions on the retention times of peaks in dAK assays 3

Table S2. X-ray crystallography data collection and refinement statistics of *G. intestinalis* dAK 4

Table S3. Cryo-EM data collection statistics of *G. intestinalis* dAK. 5

Table S4. List of interactions in the *G. intestinalis* dAK structure 6

Figure S1. Development of enzyme assay conditions for *G. intestinalis* dAK 7

Figure S2. HPLC analysis of nucleotides in the *G. intestinalis* dAK preparation 8

Figure S3. Cladribine inhibits early stages of *G. intestinalis* encystation 9

Figure S4. PI profiles from the encystation experiments 10

Figure S5. Cryo-EM methodology 11

Figure S6. *G. intestinalis* dAK particles on grids have a strong preferred orientation 12

Figure S7. Amino acid sequence alignment of *G. intestinalis* dAK with other non-TK1-like dNKs 13

Figure S8. Inhibition mechanism by dATP on *G. intestinalis* dAK 14

Figure S9. F-Ara-A selectively inhibits *G. intestinalis* proliferation 15

Figure S10. Comparison of the *G. intestinalis* dAK structure with other non-TK1-like dNKs 16

Figure S11. Unbiased F_o-F_c map showing the placement of dADP and dAMP in *G. intestinalis* dAK 17

Figure S12. *G. intestinalis* dAK Tyr73 clashes with the 2'-OH group of modelled adenosine 18

Table S1. Effect of HPLC conditions on the retention times of peaks in dAK assays. The table shows the retention times of peaks in dAK assays with deoxyadenosine analogues using isocratic conditions with a binary mixture of different ratios of solution A and B (listed as A percentage). Peaks that are independent of the A percentage are marked in grey and only listed for the 75% A protocol. Protocols with low A percentage (7 or 20%) also include elution and equilibration steps as described below.

	**HPLC protocols indicated by their mobile phase percentage of A**					
**Peak**	**7A**	**20A**	**62A**	**68A**	**75A**	**100A**
Ara-A	*Buffer-independent peak*				2.39	
dAdo	*Buffer-independent peak*				2.77	
FANA-A	*Buffer-independent peak*				3.33	
F-Ara-A	*Buffer-independent peak*				3.99	
F-dAdo	*Buffer-independent peak*				4.83	
DTT	*Buffer-independent peak*				7.70	
Cladribine	*Buffer-independent peak*				7.89	
Clofarabine	*Buffer-independent peak*				11.69	
AMP	9.01	4.46			2.68	
Ara-AMP	9.65	4.67			2.72	
dAMP	12.59	5.97			3.23	
FANA-AMP	21.35	9.43		5.11	4.53	
F-Ara-AMP	25.14	11.54			5.81	
F-dAMP	35.60	16.14		8.43	7.74	
Cladribine-MP		31.33	16.26		14.45	
Clofarabine-MP				29.46	25.38	22.98
ADP	(59.84)*	15.23	5.54	4.52	4.17	
ATP		(75.41)*	10.83	9.53	8.23	6.48
Minor peak 1			17.83	15.43	13.35	
Minor peak 2			23.05	18.70	15.74	
Minor peak 3			48.40	38.81	32.40	19.42
Analysis time	*47***	*43***	*24.2***	*19.4***	*33*	*24*

*Retention time if the isocratic conditions would be continued.
**Maximal analysis time if all listed assay products are studied. It includes the elution-equilibration procedure for the 7A and 20A protocols and is set to half the retention time of Minor peak 3 in the 62A and 68A protocols.

75A and related HPLC protocols. The 75A HPLC protocol was used for dAK assays with most deoxyadenosine analogues when tested at 200 µM (F-Ara-A, F-dAdo cladribine and clofarabine), both when assayed alone and in combination with deoxyadenosine. The samples were in these cases diluted 100 times before analysis, and the listed minor peaks are then barely visible. With more concentrated samples (5-20-fold dilutions), it was necessary to decrease the A percentage to 68% for F-dAdo and 62% for cladribine to avoid interference with the broad ATP peak and the minor peaks. The 100A protocol was used for K_M-V_{max} measurements with clofarabine to reduce the analysis time.

7A and 20A. These protocols were originally optimized for dAK assays with Ara-A, FANA-A with and without deoxyadenosine as competitor, but they are also usable for assays with most other deoxyadenosine analogies (products listed above). Both protocols require an additional 10-min step with 90% A to remove late-eluting peaks followed by a 10-min equilibration step to initial conditions. The 20A protocol gives a faster elution time for each assay product, but the 7A protocol has the advantage of a better separation between Ara-AMP and AMP.

Table S2. X-ray crystallography data collection and refinement statistics of *G. intestinalis* dAK. Statistics for the highest-resolution shell are shown in parentheses. PDBID: 8PUU.

Parameter	Result
**Wavelength (nm)**	0.976254
**Resolution range (Å)**	49.04 - 2.1 (2.175 - 2.1)
**Space group**	P 3_2 2 1
**Unit cell (a = b ≠ c, α = β ≠ γ)**	103.13 Å, 103.13 Å, 147.109 Å, 90°, 90°, 120°
**Total reflections**	1069671 (90295)
**Unique reflections**	53245 (5191)
**Multiplicity**	20 (20.9)
**Completeness (%)**	99.72 (99.12)
**Mean I/sigma(I)**	15.4 (2.6)
**Wilson B-factor**	50.25
**R-merge**	0.092 (1.396)
**R-meas**	0.097 (1.431)
**R-pim**	0.03 (1.465)
**$CC_{1/2}$**	0.999 (0.802)
**Reflections used in refinement**	53236 (5194)
**Reflections used for R_{free}**	2687 (277)
**R_{work}**	0.2000 (0.3090)
**R_{free}**	0.2247 (0.3476)
**Number of non-hydrogen atoms**	3835
**Macromolecules**	3676
**Ligands**	48
**Solvent**	111
**Protein residues**	444
**RMS (bonds, Å)**	0.017
**RMS (angles, °)**	0.91
**Ramachandran favored (%)**	98.85
**Ramachandran allowed (%)**	1.15
**Ramachandran outliers (%)**	0.00
**Rotamer outliers (%)**	0.77
**Clashscore**	3.80
**Average B-factor**	54.22
**Macromolecules (B-factor)**	54.24
**Ligands (B-factor)**	48.60
**Solvent (B-Factor**	56.08

Table S3. Cryo-EM data collection statistics of *G. intestinalis* dAK.

**Data collection**	**1**	**2**
Microscope	Titan Krios	Titan Krios
Detector	K2 (Gatan)	Falcon 4i
Energy filter slit (eV)	20	20
Voltage (kV)	300	300
Magnification	215000	215000
Objective Aperture (µm)	100	100
Specimen pixel size (Å/px)	0.63	0.58
Total dose per movie (e^-/Å^2)	59.5	60
Defocus range (µm)	-1.2 to -2.4	-0.5 to -3.5
Spherical aberration (nm)	2.7	2.7
Number of micrographs	2534	3420

Table S4. List of interactions in the *G. intestinalis* dAK structure. Detailed list of predicted interactions formed between the monomers within the crystallographic dimer of the *G. intestinalis* dAK crystal structure based on calculations using the PISA server.

Interaction #	Monomer I	Atom	Distance [Å]	Monomer II	Atom	Interaction type
**1**	M24	O	2.83	R119	NH1	Hydrogen bond
**2**	M24	O	3.76	Y132	OH	Hydrogen bond
**3**	G25	O	2.77	Y132	OH	Hydrogen bond
**4**	P29	O	3.01	R199	NE	Hydrogen bond
**5**	R119	NH2	3.26	Y28	OH	Hydrogen bond
**6**	N150	ND2	3.36	R199	O	Hydrogen bond
**7**	D156	OD2	3.31	T240	OG1	Hydrogen bond
**8**	D156	OD2	3.81	T240	N	Hydrogen bond
**9**	K198	O	2.60	Q228	NE2	Hydrogen bond
**10**	R199	O	2.92	N150	ND2	Hydrogen bond
**11**	R199	NE	3.23	P29	O	Hydrogen bond
**12**	V202	O	3.01	V231	N	Hydrogen bond
**13**	R204	NH2	2.60	P242	OXT	Hydrogen bond
**14**	R204	N	2.84	V231	O	Hydrogen bond
**15**	R204	O	2.90	V233	N	Hydrogen bond
**16**	R204	NH1	3.06	D232	OD1	Hydrogen bond
**17**	R204	NH1	3.47	A241	O	Hydrogen bond
**18**	R206	N	3.62	V233	O	Hydrogen bond
**19**	H227	O	3.12	H227	NE2	Hydrogen bond
**20**	H227	NE2	3.13	H227	O	Hydrogen bond
**21**	V231	O	2.82	R204	N	Hydrogen bond
**22**	V231	N	2.98	V202	O	Hydrogen bond
**23**	D232	OD1	2.87	R204	NE	Hydrogen bond
**24**	D232	OD2	2.96	R204	NH2	Hydrogen bond
**25**	V233	N	2.81	R204	O	Hydrogen bond
**26**	V233	O	2.95	R206	N	Hydrogen bond
**27**	D236	OD2	2.71	Y210	N	Hydrogen bond
**28**	D236	OD2	2.91	A208	N	Hydrogen bond
**29**	D236	OD1	2.95	R211	N	Hydrogen bond
**30**	D236	N	3.29	R206	O	Hydrogen bond
**31**	T240	OG1	2.55	D156	OD1	Hydrogen bond
**32**	T240	OG1	2.65	Y186	OH	Hydrogen bond
**33**	T240	N	2.89	D156	OD2	Hydrogen bond
**34**	A241	O	2.82	R204	NH1	Hydrogen bond
**35**	A241	O	2.84	R204	NH2	Hydrogen bond
**36**	A241	N	3.70	Y186	OH	Hydrogen bond
**37**	R204	NH1	3.06	D232	OD1	Salt bridge
**38**	R204	NE	3.52	D232	OD1	Salt bridge
**39**	R204	NH1	3.79	D232	OD2	Salt bridge
**40**	D232	OD1	2.87	R204	NE	Salt bridge
**41**	D232	OD2	2.96	R204	NH2	Salt bridge
**42**	D232	OD1	3.40	R204	NH2	Salt bridge
**43**	D232	OD2	3.94	R204	NE	Salt bridge

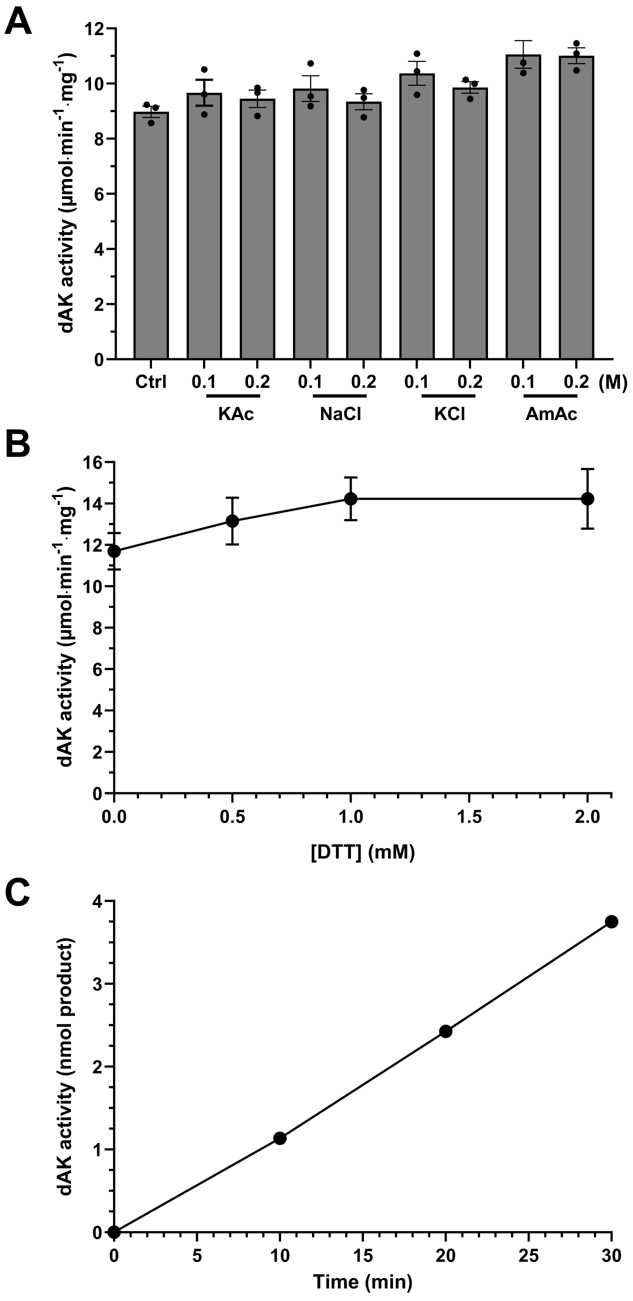

Figure S1. Development of enzyme assay conditions for *G. intestinalis* dAK. (A) Effect of different chloride and acetate salts on enzyme activity. (B) Effect of various concentrations of DTT on enzyme activity. (C) Testing the linearity of the enzyme activity over time. The enzyme assays were generally performed with 10 ng dAK, 200 µM deoxyadenosine, 2 mM ATP, 5 mM $MgCl_2$, 0.5 mM DTT (variable in B), 100 mM potassium acetate (variable salts in A) and 50 mM Tris-HCl pH 7.5 at 37°C for 30 min (variable in C). A and B represent the average of three independent experiments with standard errors indicated. A general conclusion was that the tested salts had no major effect on enzyme activity when used up to 200 mM, DTT was slightly stimulatory, and the activity was linear over 30 min.

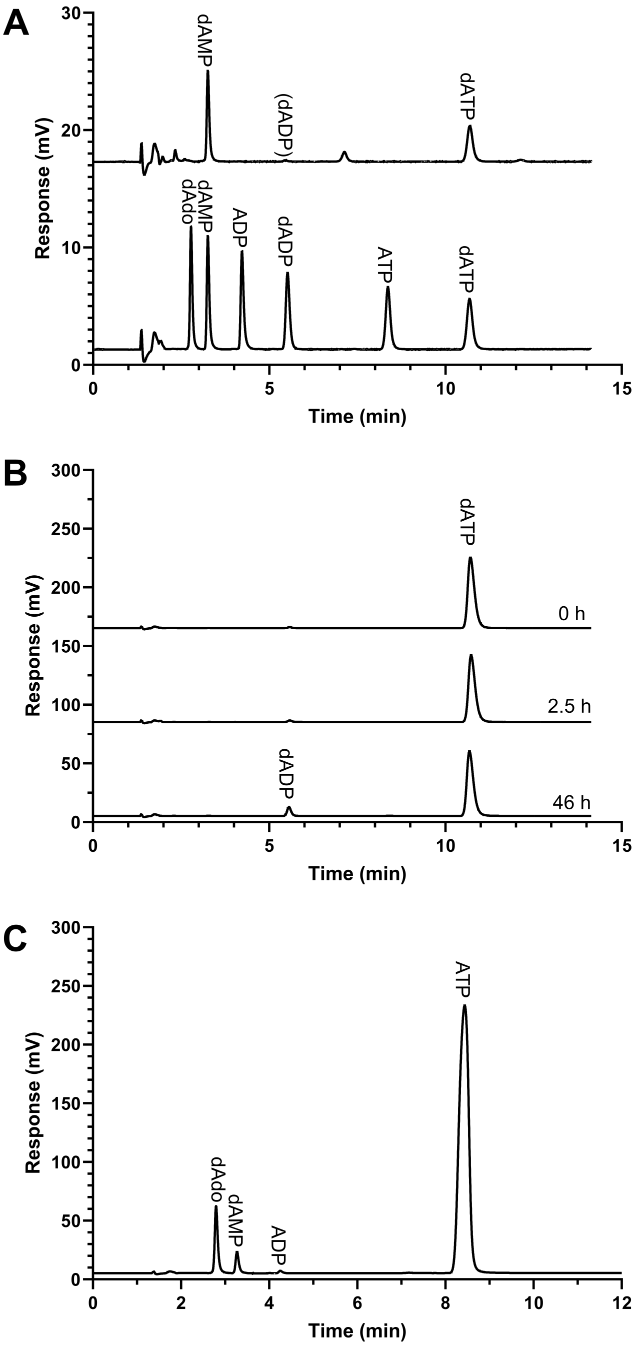

Figure S2. HPLC analysis of nucleotides in the *G. intestinalis* dAK preparation. (A) The dAK preparation contains dAMP and dATP. (B) *G. intestinalis* dAK incubated with 200 µM dATP at room temperature over 46 h shows that dATP is slowly converted to dADP. (C) Analys of dAK in a regular enzyme assay shows that this activity is much higher than the dATP-converting activity, with the latter possibly being the result of a minor impurity. The regular enzyme assay was performed with 10 ng dAK over 30 min (C) as compared to 100 ng dAK and 46 hours used in the dATP-dephosphorylation assay (B).

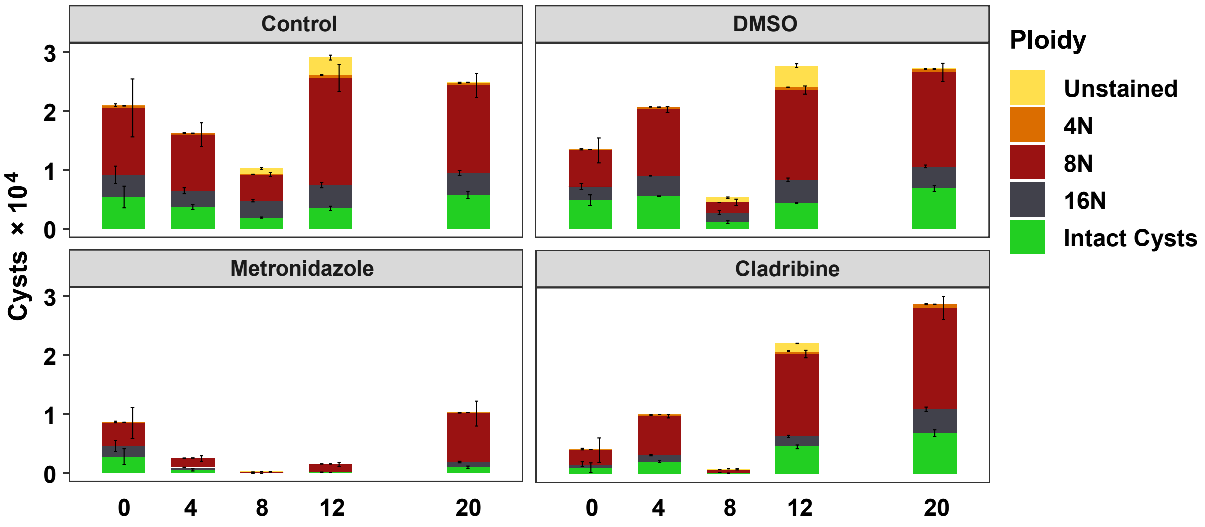

Figure S3. Cladribine inhibits early stages of *G. intestinalis* encystation. The results show the ploidy of the cells after 28 hours of incubation in encystation medium with the indicated drugs added in the beginning of the incubation or at indicated timepoints during the encystation process. The drugs were solubilized in DMSO prior to analysis. Control experiments were performed in a similar manner but with no liquid or DMSO added to the samples instead of drug. Cladribine treatment needed to be initiated within the first 12 hours to have a significant effect and then the main features were a marked reduction in the number of cells (if started within the first 8 h) and a delayed progression into 16N cells (also observable if given at the 12 h timepoint). The control cells were much less dependent on when they were treated except for the 8-h timepoint when the cells are going through a membrane reorganization process that makes them more sensitive to disturbances.

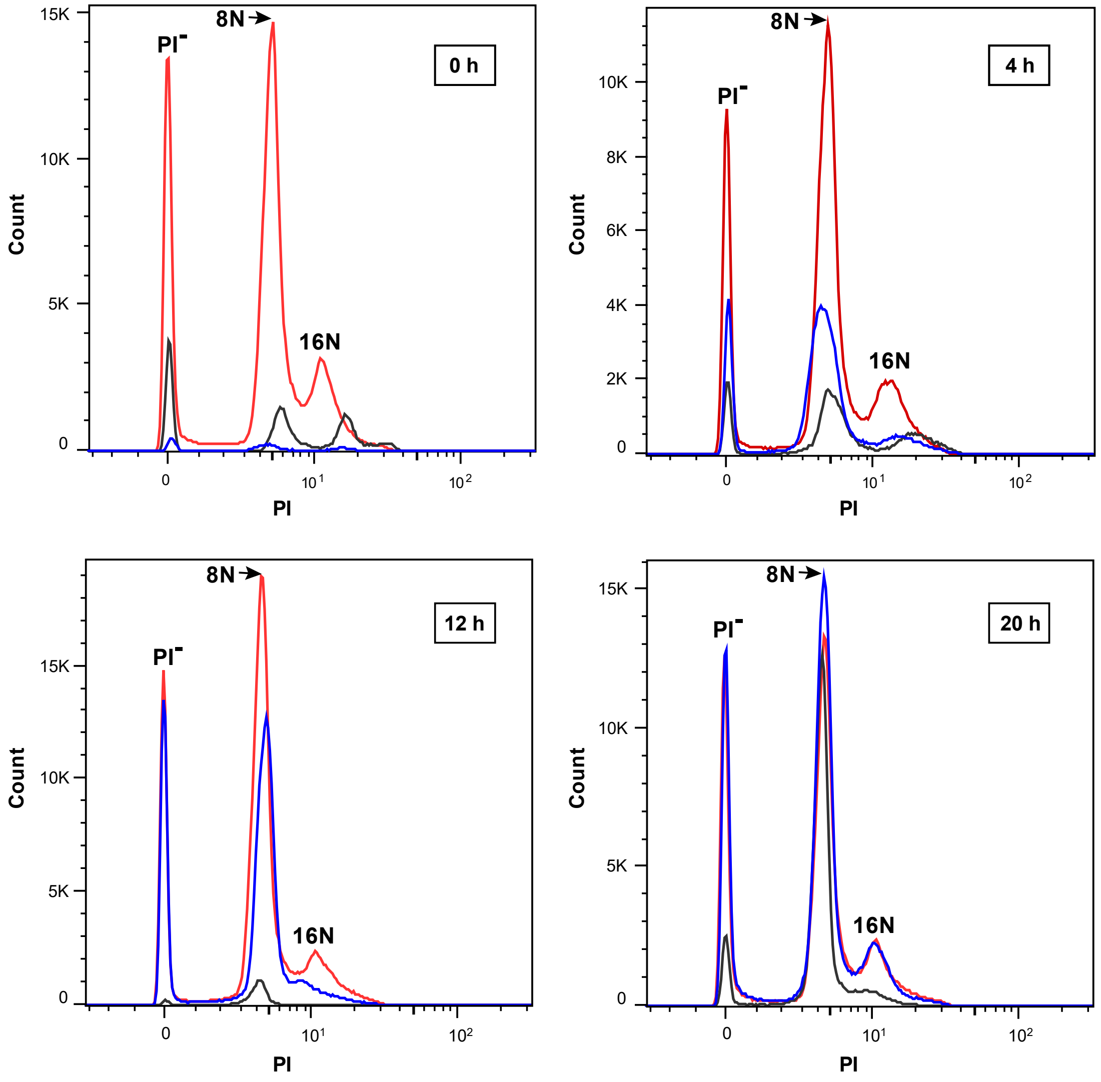

Figure S4. PI profiles from the encystation experiments. Similar type of experiment as in S5 Fig but showing the PI profile. The color code indicates control cells (red), metronidazole-treated cells (grey) and cladribine-treated cells (blue). Cladribine has a strong effect on the total number of cells if given early in the process and on the number of 16N cells if given at 12 h, but no obvious effect if given late in the process (20 h).

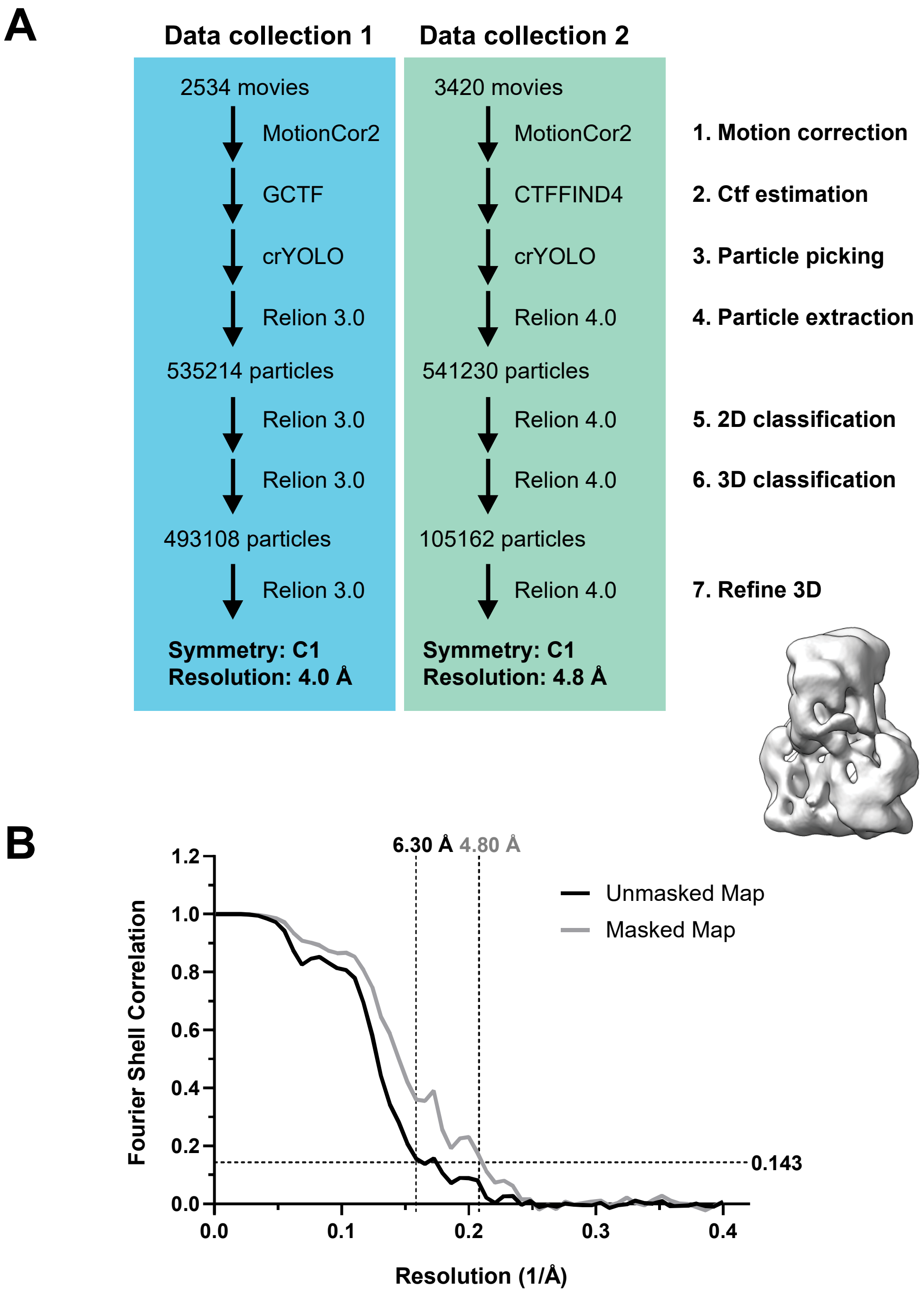

Figure S5. Cryo-EM methodology. (A) Cryo-EM data collection flowchart. (B) Graphs showing the Gold standard Fourier-Shell-Correlation (FSC) for independently refined half sets vs. resolution [1/Å] of the unmasked and masked volumes for data collection 2. FSC at 0.143 is indicated as a black dashed line. Data collection 1 generated higher quality 2D class averages and is used as the source of the images in Figure 5B. Data collection 2 generated a 3D map that looked slightly less anisotropic and is the source of the 3D map shown in Figure 5C.

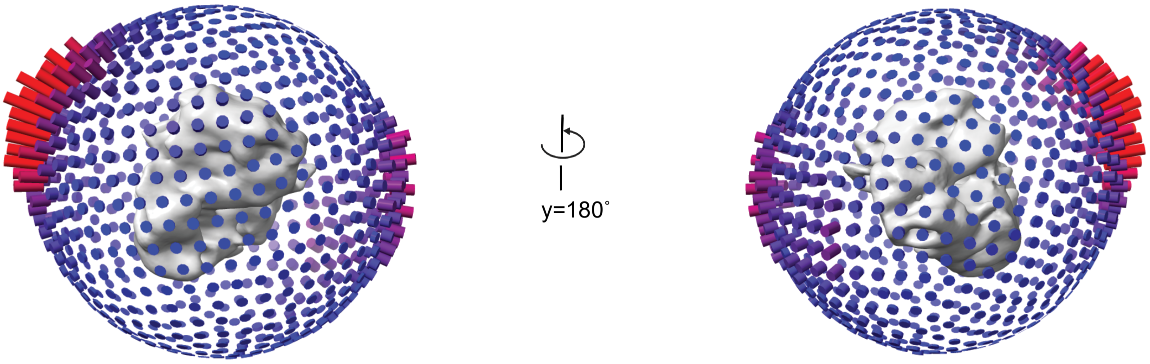

Figure S6. ***G. intestinalis*** **dAK particles on grids have a strong preferred orientation**. A frequency diagram showing the number of views from a particular direction in the dataset as the length of each bar surrounding the 3D model. The longer the red bar, the more particles present whereas purple and blue show underrepresented views with fewer particles. The image thus indicates that there are many views that are missing to build an accurate 3D model of dAK from the cryo-EM data.

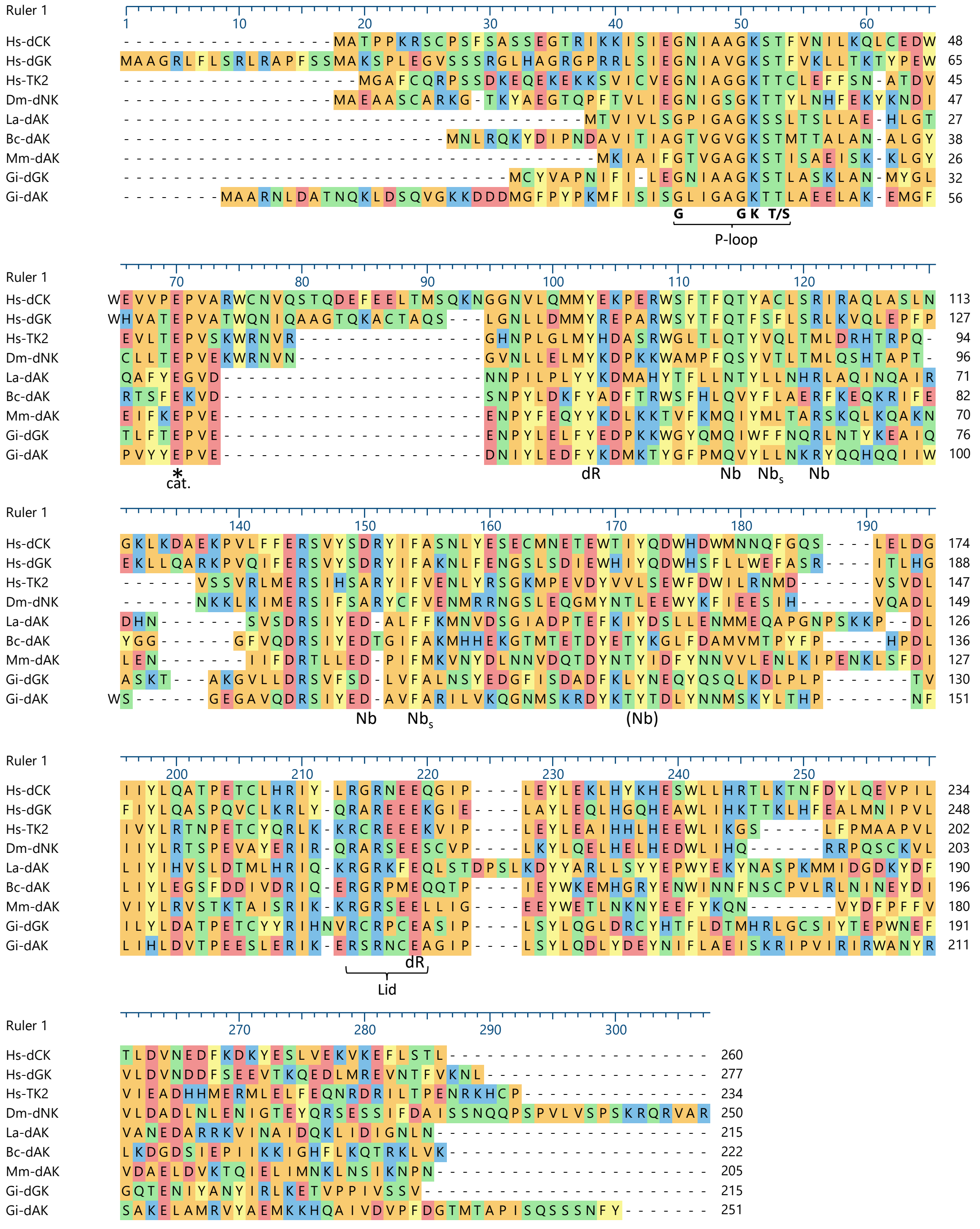

Figure S7. Amino acid sequence alignment of *G. intestinalis* dAK with other non-TK1-like dNKs. The most striking difference of the *G. intestinalis* dAK compared to the other sequences is that it has extensions in both the N-terminal and C-terminal directions. Conserved features are the P-loop for binding the phosphate donor, the catalytic base (labeled *) and the lid domain. Based on the published *Mycoplasma mycoides* dAK structure, active site residues in contact with the substrate are labeled with Nb and dR depending on if they bind the nucleobase or deoxyribose moiety. Nb$_S$ means that it is a stacking interaction with the nucleobase and brackets mean that the indicated residue affects the binding of the substrate indirectly. The alignment is performed with the Clustal W algorithm and the dNKs shown are from *Homo sapiens*, *Drosophila melongaster*, *Lactobacillus acidophilus*, *Bacillus cereus*, *M. mycoides* and *G. intestinalis*. Gi-dNK2 is the *G. intestinalis* dNK that is not studied yet.

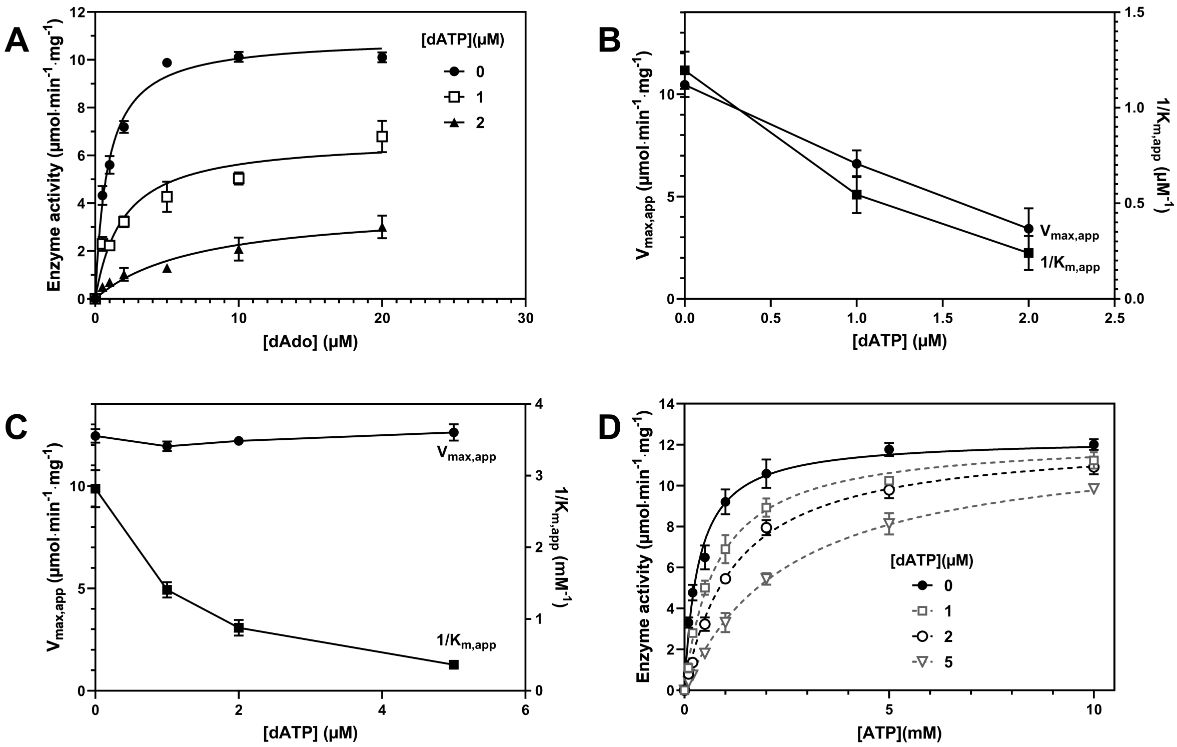

Figure S8. Inhibition mechanism by dATP on *G. intestinalis* dAK. (A) Michelis-Menten plot showing enzyme activity as a function of [deoxyadenosine] in the presence of different concentrations of dATP. $V_{max,app}$ and $K_{M,app}$ values were determined by fitting each curve to the Michaelis-Menten function by the GraphPad Prism 10.3.1 software. (B) Determined $V_{max,app}$ and $1/K_{M,app}$ values (from A) plotted as a function of dATP concentration showed that the inhibitor affects both the K_M and V_{max} values. (C) Similar plot as in B, but the $V_{max,app}$ and $1/K_{M,app}$ values was in this case obtained from experiments where ATP was the varied substrate and deoxyadenosine kept stable at 200 µM. In this case only the K_M value was affected by the inhibitor indicating competitive inhibition. (D) The data that graph C is based upon fitted to a competitive inhibition model by the software. Note that graph C is based on the same data but fitted to the regular Michaelis-Menten function to obtain $V_{max,app}$ and $K_{M,app}$ values. All graphs shown in panel A-D represent the average of three independent experiments with standard errors indicated. The experimental parameters obtained from D were the following: K_i= 0.84 ± 0.22 µM, K_M(ATP)=0.35 ± 0.05 mM, V_{max}=12.3 ± 0.2 µmol·min^{-1}·mg^{-1} and α=5.5 ± 0.9 with standard errors calculated from the variation between the individual three experiments.

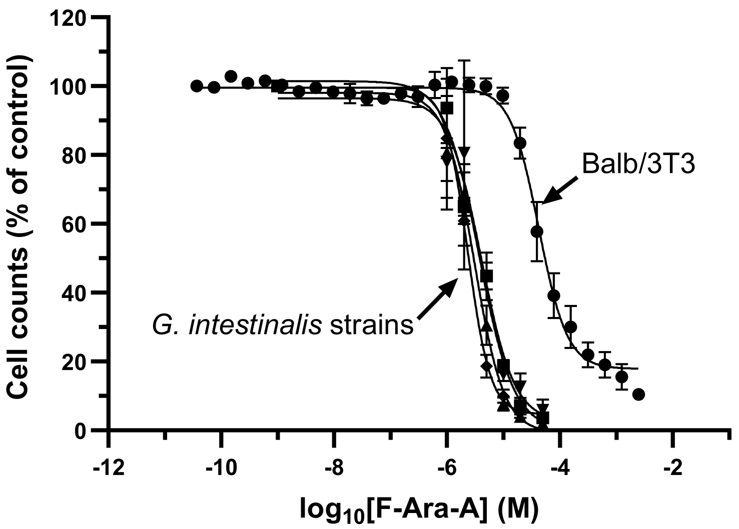

Figure S9. F-Ara-A selectively inhibits *G. intestinalis* proliferation. The figure illustrates that *G. intestinalis* is more sensitive than the mammalian reference cell line to F-Ara-A and that the parasite strains are equally affected by the drug regardless of if they are metronidazole-resistant or not. The cells used in the experiments are mammalian Balb/3T3 fibroblasts (●), the *G. intestinalis* parent strain WB (■), the metronidazole-resistant strains M1 (▲), the metronidazole-resistant strain M2 (▼), and the resistant revertant strain M1-NR (◆). The graphs represent the average of 3 or more experiments with standard errors and the data is fitted to a log [inhibitor] vs response (variable slope) curve by GraphPad Prism 10.1.0.

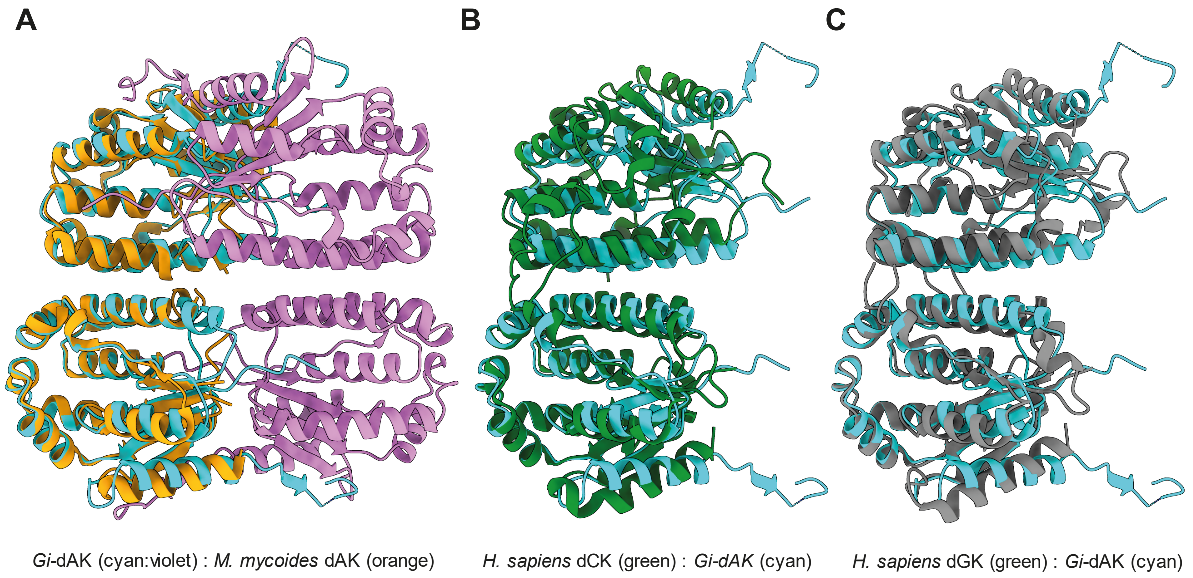

Figure S10. Comparison of the *G. intestinalis* dAK structure with other non-TK1-like dNKs. Although the structural fold of the polypeptides is similar in the three examples, *G. intestinalis* dAK (labeled *Gi*-dAK) sticks out by being tetrameric. Note that it is only in A that the whole tetramer is shown, whereas B-C is focused on the dimer part to highlight the homology. (A) Tetrameric structure of *G. intestinalis* dAK (cyan/magenta) superimposed onto the structure of *M. mycoides* dAK (orange, PDBID: 2JAQ). (B) *G. intestinalis* dAK dimer (cyan) superimposed onto *H. sapiens* dCK (green, PDBID: 2ZI3). (C) *G. intestinalis* dAK dimer (cyan) superimposed onto *H. sapiens* dGK (grey, PDBID: 2OCP). Protein structures are shown as cartoon representation.

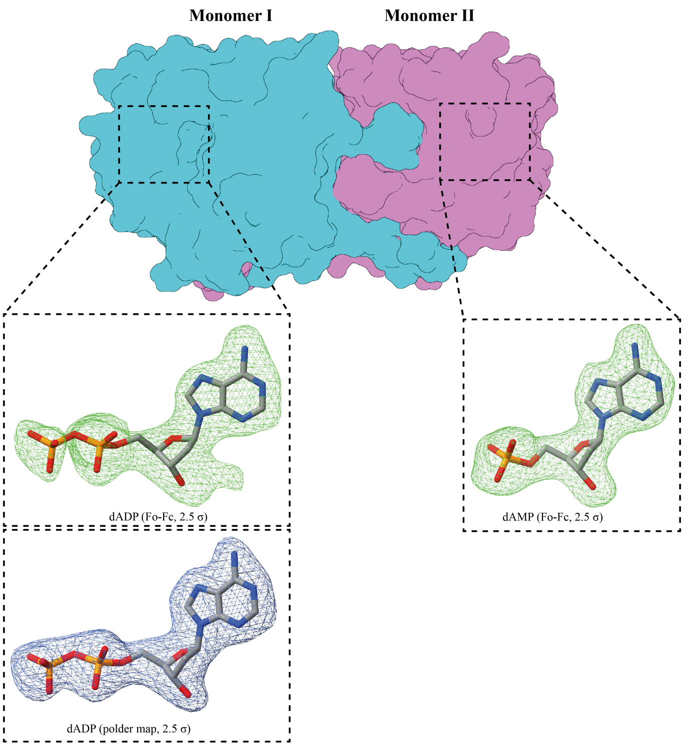

Figure S11. Unbiased F_o-F_c map showing the placement of dADP and dAMP in *G. intestinalis* dAK. The crystallographic dimer of *G. intestinalis* dAK revealed that two ligands were co-purified during protein purification. Monomer I (cyan) contains density belonging to a dADP molecule, with a partial (0.5) occupancy for the b-phosphate (indicating a mixture of dADP and dAMP), and monomer II (magenta) contains density for a dAMP molecule. dAMP and dADP are shown as grey sticks, the unbiased *Fo-Fc* density map as a green mesh, and the dADP polder map as blue mesh. All meshes were contoured to 2.5 σ.

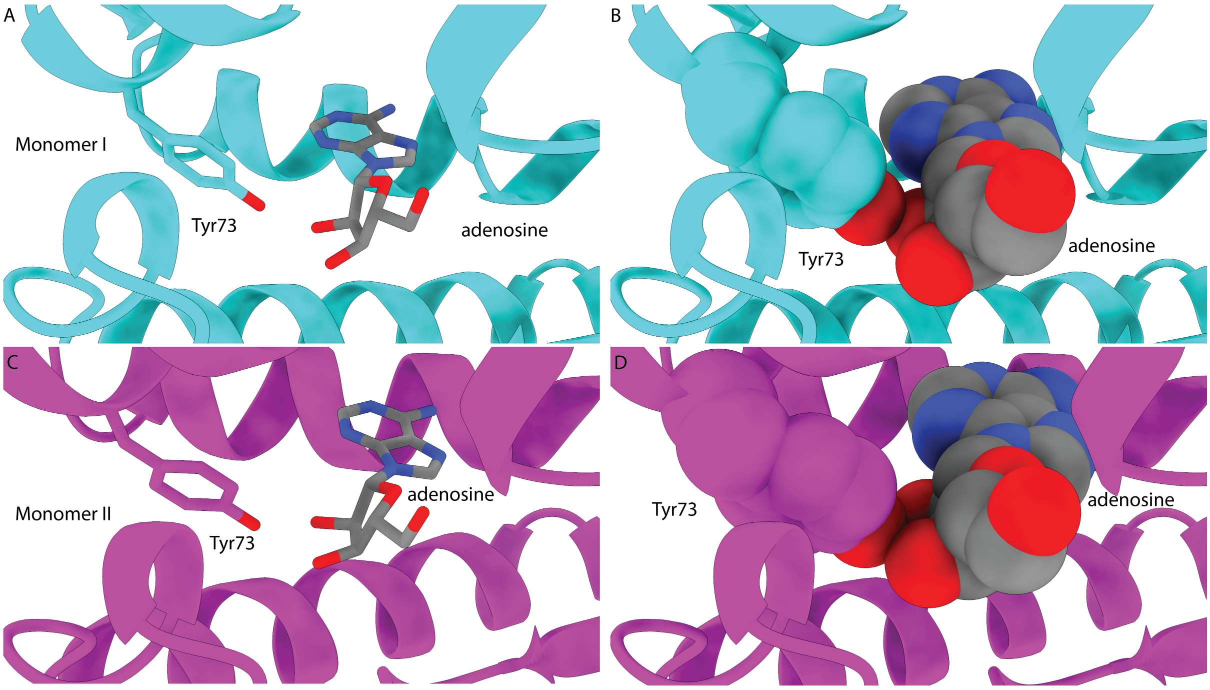

Figure S12. ***G. intestinalis*** **dAK Tyr73 clashes with the 2'-OH group of modelled adenosine.** Structures showing a modelled adenosine molecule in the mixed dADP/dAMP site (monomer I) and the dAMP site (monomer II). The left figures show cartoon representations of the sites with Tyr73 and adenosine indicated as sticks. In the right figures, Tyr73 and adenosine (grey) are shown as spheres corresponding to their individual Van der Waals radii. (A) + (B) Monomer I, (C) + (D) Monomer II.
